# Smart Hydrogel Doped by Metal–Organic Frameworks for Renewable Self‐Pumping Enzymatic Reactors

**DOI:** 10.1002/advs.202507886

**Published:** 2025-11-29

**Authors:** Yuxi Zhu, Shenghao Wang, Yueyuan Luo, Linbo Cao, Xiuxiu Guo, Yue Zhang, Mingmin Li, Tie Wang

**Affiliations:** ^1^ Tianjin Key Laboratory of Life and Health Detection Life and Health Intelligent Research Institute Tianjin University of Technology Tianjin 300384 P. R. China

**Keywords:** active diffusion, enzyme immobilization, MOFs, regeneration, smart hydrogel

## Abstract

The construction of high‐performance immobilized enzymes is vastly desired for green biotransformation. Although hydrogels offer significant potential for facilitating biomedical applications of enzymes due to their flexibility, macroscopic processability, and extracellular matrix‐like properties. However, maintaining enzymatic activity and recyclability within bulk hydrogels remains a formidable challenge due to restricted passive mass transfer and enzyme leakage. Herein, inspired by the heart's blood‐pumping mechanism, a smart metal–organic framework (MOFs)‐doped hydrogel is developed for enzyme immobilization to effectively address these challenges. Importantly, thermally triggered contraction/expansion cycles of the hydrogel, coupled with a dynamic water stream, significantly enhance substrates intake and endogenous products expulsion, while enzymes are effectively retained due to the interception effect of MOFs. This activity can be tuned by adjusting hydrogel contractility, enabling active mass transfer and regeneration similar to living tissues. Benefit from these advantages, the biosystems perform well in biosensing applications. This study provides a novel solution for enzymatic immobilization in porous 3D matrices and opens opportunities for the construction of life‐like bio‐microdevices or artificial organelles.

## Graphical abstract

Renewable self‐pumping enzymatic reactors based on thermo‐stimulated cyclic contraction/expansion of metal‐organic frameworks‐doped hydrogel.

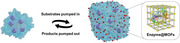

## 1 Introduction

Biocatalysis occurs commonly in nature and plays a vital role in maintaining normal life activities.^[^ ^1^, ^2^, ^3^ ^]^ Benefiting from their environmental friendliness, high efficiency, and selectivity, the construction of cell‐free enzymatic catalysis in vitro has attracted significant attention in biotechnology.^[^ ^4^, ^5^, ^6^ ^]^ However, their fragile nature and poor recyclability greatly hinder their industrial application.^[^ ^7^, ^8^, ^9^, ^10^ ^]^ Immobilization technology is a facile and efficient strategy for improving enzyme tolerance, reusability, and selectivity.^[^ ^11^, ^12^, ^13^, ^14^, ^15^, ^16^ ^]^ Different from nano‐/micropower, the construction of enzymatic reactors based on processable macrostructures is more practical. Recently, unique three‐dimensional cross‐linked hydrophilic networks of hydrogels have endowed them with superior potential for mimicking the mechanical and biological functions of natural tissues.^[^ ^17^, ^18^, ^19^, ^20^ ^]^ This kind of material can provide an extracellular matrix (ECM)‐like environment in addition to protecting the entrapped enzymes, thus offering new applications for immobilized enzymes in soft robotics, artificial organs, and wearable/implantable devices.^[^ ^21^, ^22^, ^23^, ^24^ ^]^ Despite their importance, enzyme‐immobilized hydrogels remain underutilized in large‐scale industrial applications. The restricted passive diffusion process driven by the concentration gradient in bulk hydrogels results in a delayed response to substrates, as well as a serious accumulation of products. These biocatalytic systems exhibit poor activity and reproducibility, and require disposability in most cases, increasing the economic cost.^[^ ^25^, ^26^, ^27^ ^]^ Therefore, the fabrication of novel strategies to realize the rapid reversible mass exchange of bulk hydrogels with the external environment and long‐lasting enzyme retention, seemingly mutually exclusive, is critical and appealing in various fields.

**Scheme 1:**
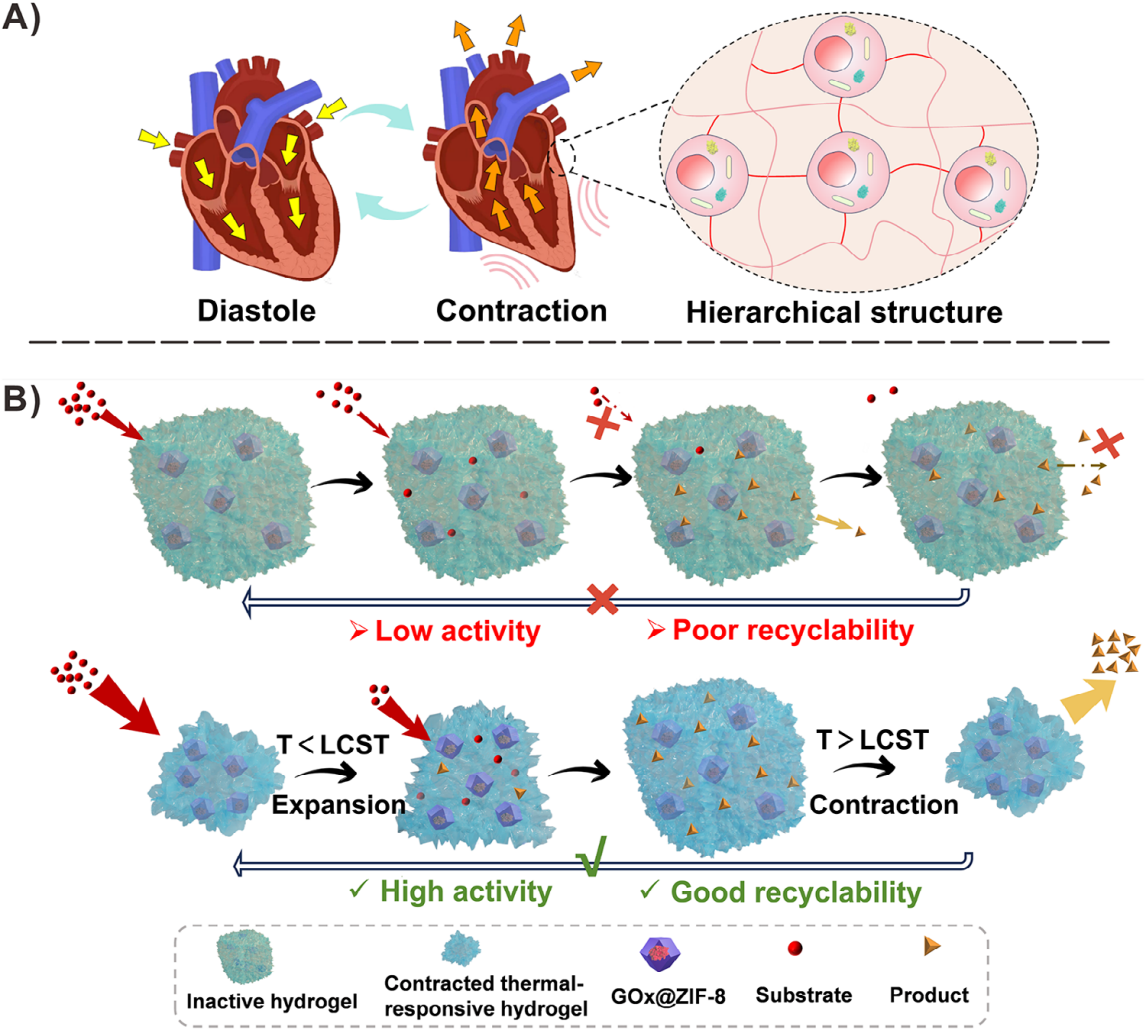
A) The diagram of the sustainable contraction/diastole of the heart muscle and blood pumping in/out based on hierarchical tissue structure. B) Schematic illustration of the passive mass exchange promoted by concentration difference (upper) and the active mass exchange promoted by thermo‐stimulated cyclic contraction/expansion of MOFs‐doped hydrogel (lower).

The structures of catalytic organisms in nature, which have advanced active transport abilities to sustain normal life activities, may provide an ingenious and feasible approach for overcoming these challenges. In particular, contraction and diastole of the heart muscle generate a pressure gradient that offers energy for blood pumping, thus allowing an efficient supply of nutrients and elimination of waste.^[^ ^28^, ^29^ ^]^ Smart hydrogels can respond to specific external stimuli and undergo dramatic volume changes or hydrogel‐to‐solution phase transitions, which enable the precise regulation of bioreactor behaviors in immunomodulation, actuators, and biosensing possible.^[^ ^30^, ^31^, ^32^, ^33^ ^]^ In particular, hydrogel matrices with ultrafast and high‐ratio swelling/shrinking capability have been developed and demonstrated to significantly enhance the transport and transformation of biomolecules.^[^ ^34^, ^35^ ^]^ However, enzyme leakage is still inevitable. Remarkably, enzymes are often hierarchically organized in tissues (**Scheme** **1**A).^[^ ^36^, ^37^ ^]^ This provides protection for the biocatalysts and prevents leakage during metabolism. Metal‐organic frameworks (MOFs) have been proven to be a kind of promising candidate to efficiently encapsulate single or multi enzymes for cell‐like bioreactions.^[^ ^38^ ^]^ Their ultrahigh surface area, well‐defined porosity, as well as readily engineered functionality, also endow them with great potential to construct particle‐in‐hydrogel soft intelligent biomaterials, and realize active mass exchange and cyclic catalysis, which, however, is still basically unexplored.

Inspired by nature, this study fabricated a smart platform based on MOFs‐doped hydrogel to immobilize enzymes, which demonstrated the rapid response and regeneration of this novel macroscopic enzymatic reactor (Scheme 1B). Importantly, the thermally triggered switch between hydrophilic/hydrophobic properties, accompanied by cyclic expansion/contraction of the hydrogel, could act as a water pump. This pump effect facilitates the capture and concentration of substrates around enzymes during the reaction. Additionally, the products were actively expelled from the matrix after the reaction. The interior enzymes were well‐preserved because of the trapping effect of the MOFs. The above characteristics define a highly stable and reusable catalytic ability of the systems due to the synergistic protection provided by both MOFs and hydrogel. Moreover, this strategy is highly versatile, allowing customization for a wide range of single‐enzyme and multi‐enzyme catalytic reactions applicable to various fields, such as biosensing.

## 2 Results and Discussion

### 2.1 Preparation and Characterization of Thermal Responsive Bio‐hydrogel Composite

Zeolitic imidazole frameworks (ZIFs) are promising candidates for integration with hydrogels and the construction of hierarchical biocatalytic systems, as they efficiently encapsulate enzymes in situ, regardless of size.^[^ ^38^, ^39^ ^]^ In this study, glucose oxidase immobilized in ZIF‐8 (GOx@ZIF‐8) was prepared to demonstrate this concept. Powder X‐ray diffraction (PXRD) showed that GOx incorporation did not affect ZIF‐8 crystallinity (Figure S1, Supporting Information). Fourier transform infrared (FT‐IR) spectroscopy confirmed the successful integration of GOx into ZIF‐8 (Figure S2, Supporting Information). Transmission electron microscopy (TEM) images revealed increased surface roughness of the enzyme‐loaded material compared to pure ZIF‐8 (Figure S3, Supporting Information). The GOx loading amount was determined to be 79 mg g^−1^ using the Bradford assay (Figure S4, Supporting Information).

**Figure 1:**
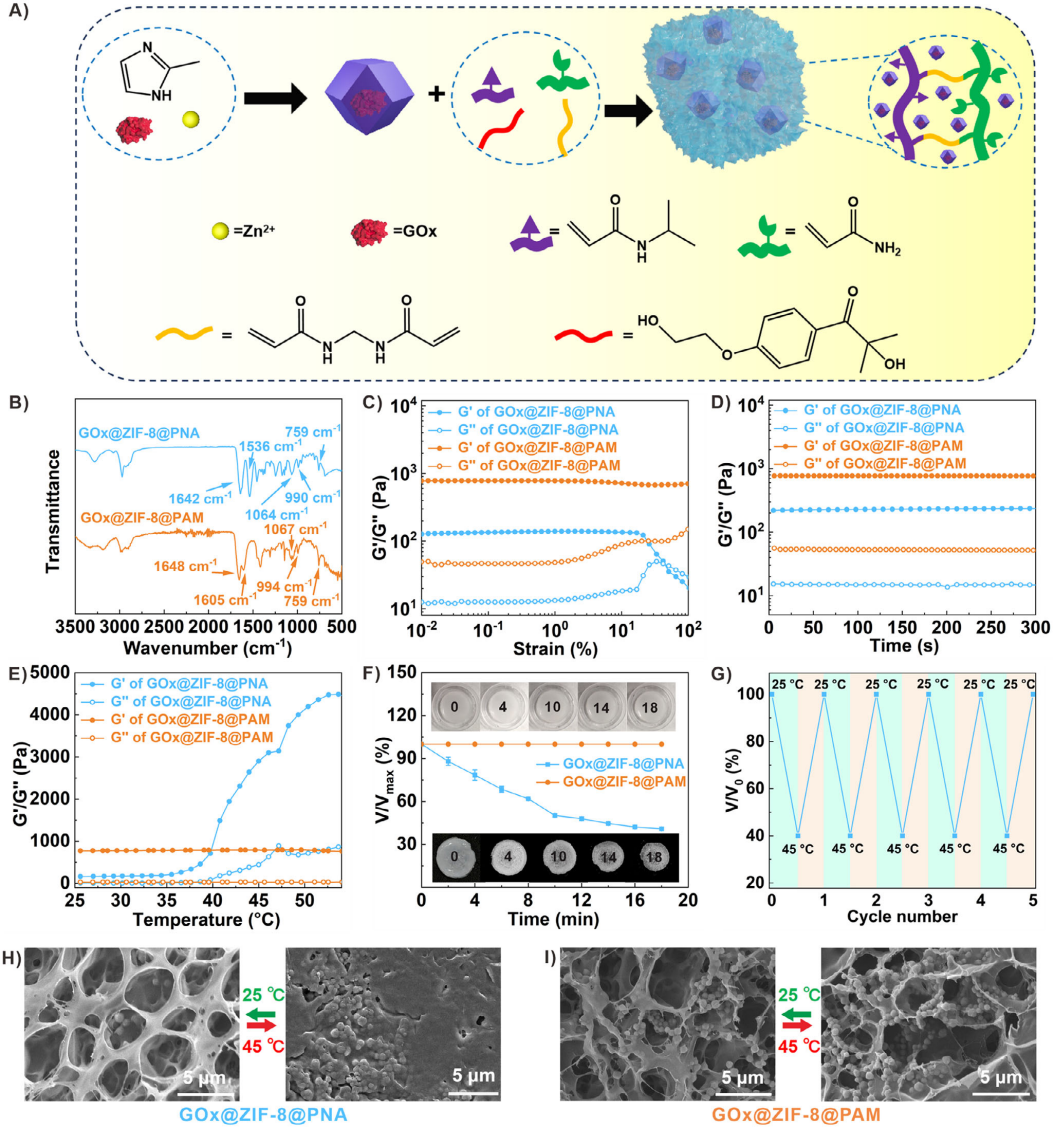
Synthesis and basic characterizations of thermal‐responsive GOx@ZIF‐8@PNA. A) Schematic illustration of fabricating GOx@ZIF‐8@PNA. B) FT‐IR spectra of GOx@ZIF‐8@PNA and GOx@ZIF‐8@PAM. C) The oscillating strain scanning curves of GOx@ZIF‐8@PNA and GOx@ZIF‐8@PAM at a fixed frequency of 1 Hz. D) Oscillation time scanning of GOx@ZIF‐8@PNA and GOx@ZIF‐8@PAM at 1 Hz and 1% strain. E) Rheological measurements at different temperatures illustrated the thermal responsive property of GOx@ZIF‐8@PNA and the inactive property of GOx@ZIF‐8@PAM gel. F) Dynamic volume change of GOx@ZIF‐8@PNA and GOx@ZIF‐8@PAM with the increase of heating time. G) Reversible volume change of GOx@ZIF‐8@PNA gel in response to temperature. SEM images of partial region of H) GOx@ZIF‐8@PNA and I) GOx@ZIF‐8@PAM at 25 and 45 °C.

Poly(*N*‐isopropylacrylamide) (PNIPAM) enables active reversible mass exchange with the external environment by its mild crosslinking pathway, good biocompatibility, and pronounced thermal response at near physiological temperature (lower critical solution temperature, LCST = 32.5 °C). In this study, PNIPAM was prepared using a previously reported free‐radical photocuring method with modifications.^[^ ^40^ ^]^ To strengthen its mechanical properties, a small portion of acrylamide (AM), was added, resulting in PNIPAM‐PAM (denoted as PNA, *n*_NIPAM_: *n*_AM_ = 24.5:1) (Figures S5 and S6, Supporting Information). Inactive PAM was used as a control. The obtained GOx@ZIF‐8 was then incorporated into the hydrogel preparation to facilitate chemical crosslinking of the polymer components around ZIF‐8 (**Figure** **1**A). FT‐IR spectra showed (Figure 1B; Figure S7, Supporting Information) characteristic peaks from both GOx@ZIF‐8 (994/990 cm^−1^, 759 cm^−1^) and the hydrogels (1648, 1642, 1605, 1536 and 1067/1064 cm^−1^), indicating successful combination. Oscillatory rheological measurements were performed to study the viscous and elastic properties of the hydrogel composites (Figure 1C,E; Figure S8, Supporting Information). Similar to PNA and PAM, oscillatory strain scanning of GOx@ZIF‐8@PNA and GOx@ZIF‐8@PAM revealed a linear viscoelastic (LVE) region, where the storage modulus (G′) was significantly higher than the loss modulus (G′′), validating the generation of a solid hydrogel network. An oscillation‐time scan confirmed the stability of the composite hydrogels. The LCST of PNA and GOx@ZIF‐8@PNA are about 35.9 °C, calculated by oscillation in a continuous temperature change test, which were slightly higher than that of pure PNIPAM (32.5 °C) (Figure S9, Supporting Information). Both values are permitted for enzyme stability. At increased temperatures, the hydrogel volume of GOx@ZIF‐8@PNA decreased to 40% of the original value as incubation time increased (Figure 1F). This result was accompanied by a large amount of water being squeezed out. The G′ /G″ values and the volume of GOx@ZIF‐8@PAM minimally changed with increased temperature. GOx@ZIF‐8@PNA exhibited a significant reversible contraction/expansion behavior through variable‐temperatures (Figure 1G). Scanning electron microscopy (SEM) images depicted freeze‐dried expansive PNA, and GOx@ZIF‐8@PNA contained macroporous channels, which collapsed in the contracted state (Figure 1H; Figure S10, Supporting Information). Both for expanded and contracted states, ZIF‐8 was uniformly distributed in the hydrogel (Figure S11, Supporting Information). The macropores of PAM and GOx@ZIF‐8 @PAM were retained after heat treatment (Figure 1I and Figure S12, Supporting Information). This thermally initiated volume conversion of PNA provides a platform for substrate pumping in and product pumping out.

### 2.2 Thermal‐Induced Pump‐in/out Performance of PNA

**Figure 2:**
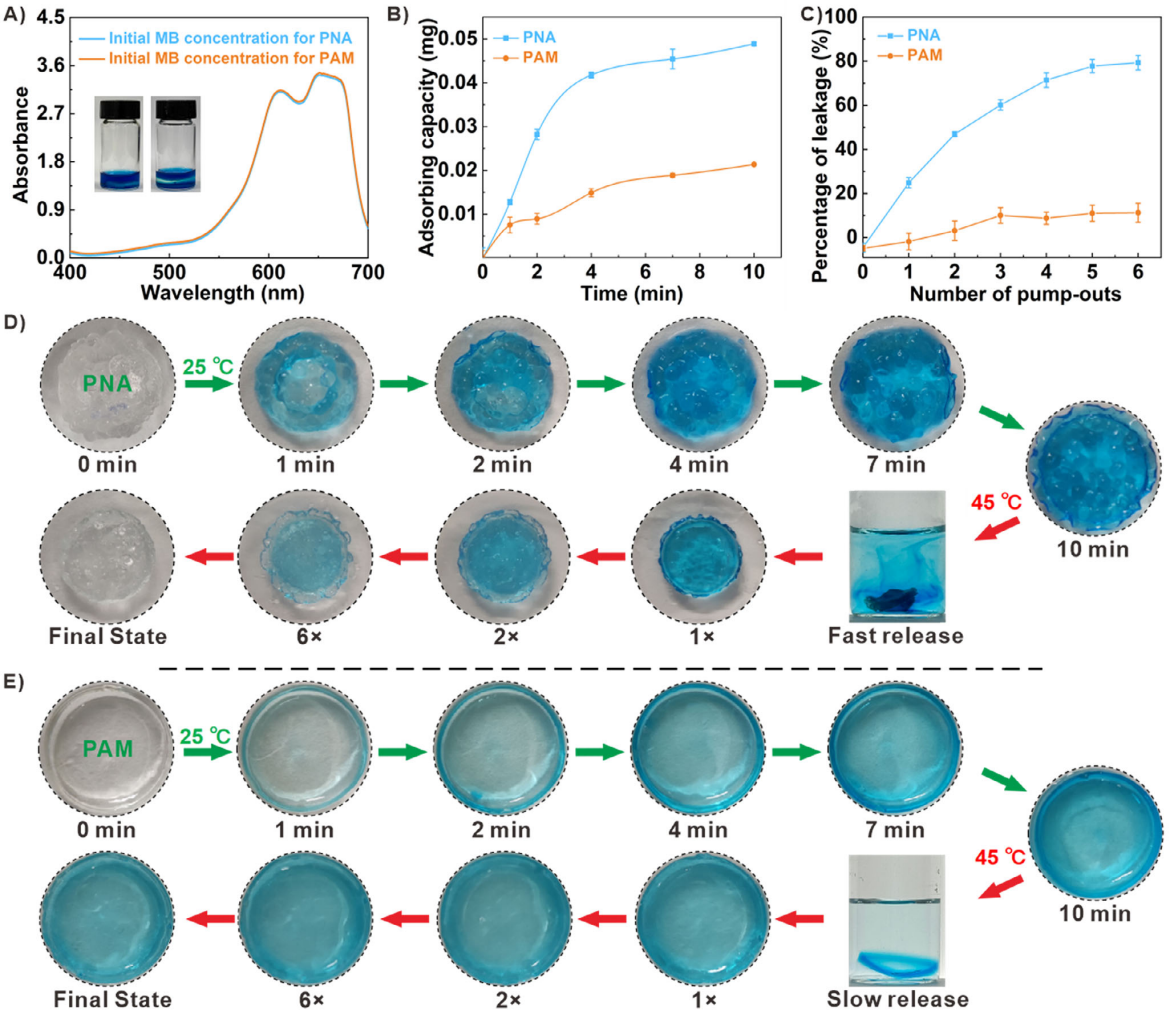
Pump‐in and regeneration behavior of thermal‐responsive PNA. A) UV–Vis spectra of methylene blue (MB) solution used for PNA and PAM adsorption. The insets are photo images of immersed PNA and PAM in the initial state, respectively. B) The adsorption curves of PNA and PAM toward MB at different time intervals. C) The release curves of PNA and PAM toward MB after different pump‐out numbers. Photographs of D) PNA and E) PAM during the process of methylene blue adsorption (25 °C) and release (45 °C) cycle.

Water‐soluble methylene blue (MB) was used as a model molecule to validate the active substrate diffusion process of PNA, with non‐heat‐responsive PAM immersed in the dye solution at the same concentration as the control (**Figure** **2**A; Figure S13, Supporting Information). UV–Vis spectroscopy and fluorescence imaging demonstrated that both the adsorption rate and amount of contracted PNA were higher than those of PAM (Figure 2B; Figure S14, Supporting Information). When the MB‐loaded hydrogels were treated at 45 °C, faster MB release from PNA was observed, accompanied by volume contraction (the release percentage can reach up to 80% in 6 times of contraction/expansion cycles, which was only 18% in PAM through passive diffusion (Figure 2C). In Figure 2D,E, the contracted PNA exhibited significantly faster uptake of the external dye during its expansion process compared to PAM. Upon thermal treatment, dye‐loaded PNA showed clear color flows from the hydrogel to the external solution within 2 min and could return close to its original state through repeated contraction/expansion cycles. In contrast, PAM remained dark blue after the same treatment. These results demonstrate that thermally triggered reversible contraction/expansion processes effectively facilitate the formation of dynamic water streams, enhancing substance exchange between the hydrogel matrix and the surrounding solution.

### 2.3 Catalytic Performance of GOx@ZIF‐8@PNA

**Figure 3:**
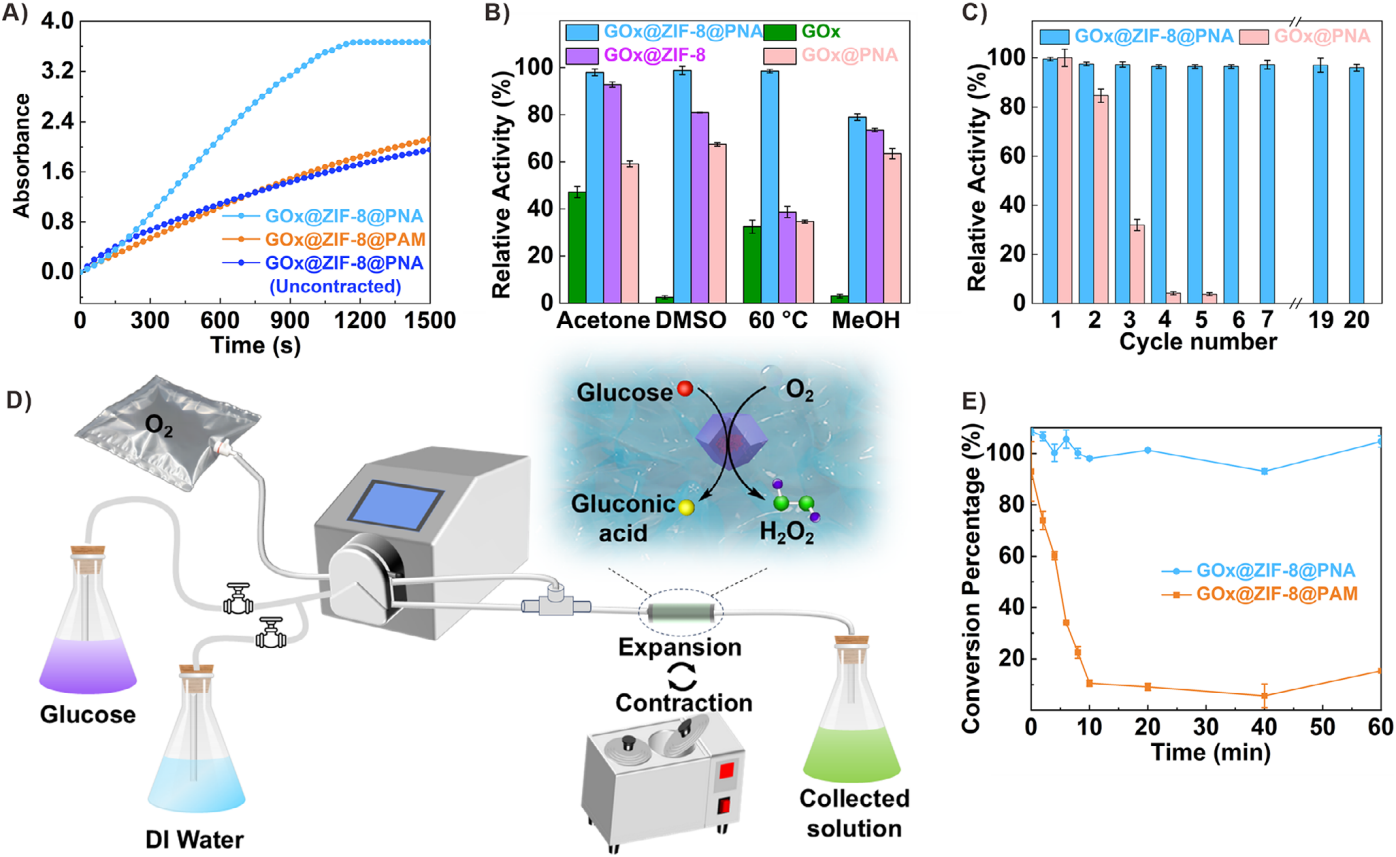
Biocatalytic performance of GOx@ZIF‐8@PNA. A) Catalytic activity contrast of GOx@ZIF‐8@PNA, initial expanded GOx@ZIF‐8@PNA, and GOx@ZIF‐8@PAM. B) Activity percentage of GOx, GOx@ZIF‐8, GOx@PNA, and GOx@ZIF‐8@PNA after being treated under various harsh conditions. C) Recycling experiments of GOx@PNA, GOx@ZIF‐8@PNA. D) Flow chart images of the glucose continuous conversion device. E) Conversion percentage of glucose for GOx@ZIF‐8@PAM and GOx@ZIF‐8@PNA at various times.

The cascade reaction method was used to measure the catalytic activities of the bio‐hydrogels. As GOx spontaneously oxidizes glucose to gluconic acid, while producing H_2_O_2_, it can serve as the substrate for HRP in the presence of ABTS, generating a strong colorimetric signal (ABTS^+^). All substrates and conditions exhibited little effect on GOx activity (Figures S15–S17, Supporting Information). By tracking the absorbance change at 418 nm, the contracted GOx@ZIF‐8@PNA had significantly higher activity than the expansive GOx@ZIF‐8@PNA, which exhibited no significant difference in activity from GOx@ZIF‐8@PAM (with the same amount of enzyme and catalytic temperature) (**Figure** **3**A). However, compared with free enzymes, the immobilized system exhibited lower absolute activity, representing a reasonable trade‐off for improved stability and reusability (Figure S18, Supporting Information). Subsequently, the activity of GOx@ZIF‐8@PNA was evaluated across a range of temperatures to further refine operational conditions. The results revealed that the bio‐hydrogels achieved maximum performance at 25 °C (Figure S19, Supporting Information). At lower temperatures, substrate pumping efficiency may increase; however, this is accompanied by a reduction in catalytic activity (Figures S20 and S21, Supporting Information). Therefore, 25 °C was determined to be the optimal temperature that balances both catalytic performance and substrate pumping efficiency. Additionally, the product expulsion efficiency was investigated at various temperatures above the LCST to optimize the self‐cleaning capability. Using MB as a model substrate, results indicated that the efficiency improves with increasing temperature (37, 41, and 45 °C). Nevertheless, at excessively elevated temperatures (e.g., 50 °C), pronounced hydrogel contraction results in the entrapment of residual products within the bulk matrix, thereby compromising cleaning efficacy (Figure S22, Supporting Information). Consequently, 45 °C was determined to be the optimal temperature for subsequent applications. By increasing the heating time, the degree of hydrogel contraction can be controlled, further optimizing the activity by increasing the water pumping strength (Figure S23, Supporting Information). These results demonstrate that the active transfer of the substrate caused by volume expansion can effectively promote contact between the substrate and enzyme, improving catalytic efficiency.

The tolerance of GOx@ZIF‐8@PNA was examined under various perturbation conditions, including DMSO, acetone, MeOH, and high temperature. GOx, GOx@ZIF‐8, and GOx@PNA were selected as the control groups. As shown in Figure 3B, the activity of free GOx decreased sharply after all treatments. ZIF‐8 and PNA were seen to offer a certain degree of protection toward GOx (retaining 92.8% and 59.1% of the original activity in acetone, the values were 80.9% and 67.4% in DMSO, 38.7% and 34.6% at 60 °C, 73.5% and 63.5% in MeOH, respectively). In contrast, the activity of GOx@ZIF‐8@PNA was preserved at 98.0%, 98.8%, 97.0%, and 79.0%, respectively. This indicates that PNA and ZIF‐8 can provide dual protection to GOx, improving its environmental tolerance. As repeated heating is necessary for recyclability, the catalytic activity was further measured during continuous exposure of the hybrid enzyme to a fixed temperature of 60 °C to assess thermal stability. The results showed that the activity of GOx@ZIF‐8@PNA was negligibly affected, despite continuous heating for 2 h, differing from that of free GOx (Figure S24, Supporting Information).

The durability of the obtained catalytic system, an important criterion for heterogeneous biocatalysts, was also investigated. As shown in Figure 3C, the activity of GOx@ZIF‐8@PNA was readily renewed for at least 20 cycles, with negligible leakage detected during the recycling experiments (Figure S25, Supporting Information). In contrast, free GOx, being soluble in the reaction system, cannot be recovered after reaction and thus has no recyclability at all. Meanwhile, GOx@PNA without ZIF‐8 exhibited no recyclability due to GOx leakage during heating. This demonstrates that the retention effect of ZIF‐8 effectively seals the enzyme within the hydrogel, allowing only water‐soluble small molecules to be pumped out during the switching cycles. In addition, system recovery is easily achieved, in contrast to powder‐based immobilization platforms (e.g., ZIF‐8), where centrifugation is required, and enzymes are prone to loss despite careful handling (Figure S26, Supporting Information). So, we can conclude that ZIF‐8 and heat‐responsive PNA cooperate and complement each other to regenerate the catalytic system.

The enhanced renewability, processability, and stability of the prepared biological hydrogel components enabled the construction of a device to assess its catalytic performance in a continuous‐flow reaction. This approach offers advantages for industrial‐scale production over traditional batch models, where the high solubility of free enzymes or suboptimal immobilization efficiency causes inevitable enzyme leakage and substantial product accumulation, leading to unsustainable operation and requiring continuous enzyme supplementation to sustain activity—an economically and operationally unfeasible demand for industrial‐scale applications. Figure 3D illustrates the flowchart and digital images of the continuous glucose conversion device. During the catalytic process, repeated expansion‐contraction operations were applied to regenerate the system. The outlet solution was collected at specific time intervals to quantify the glucose concentration (DNS method, Figure S27, Supporting Information) and measure the conversion rate. Impressively, GOx@ZIF‐8@PNA with brake‐diffusion property maintained an enduring high catalytic efficiency (conversion percentage of ≈100%) over an extended period (60 min) (Figure 3E). As a control experiment without the active pumping effect, GOx@ZIF‐8@PAM exhibited a high conversion rate at the beginning, followed by a sharp decrease with the accumulation of the product, preventing the contact of glucose with the catalyst. These results demonstrate the advantages of the MOF‐hydrogel catalytic system in a continuous reaction apparatus.

### 2.4 The Exploration of Universality

**Figure 4:**
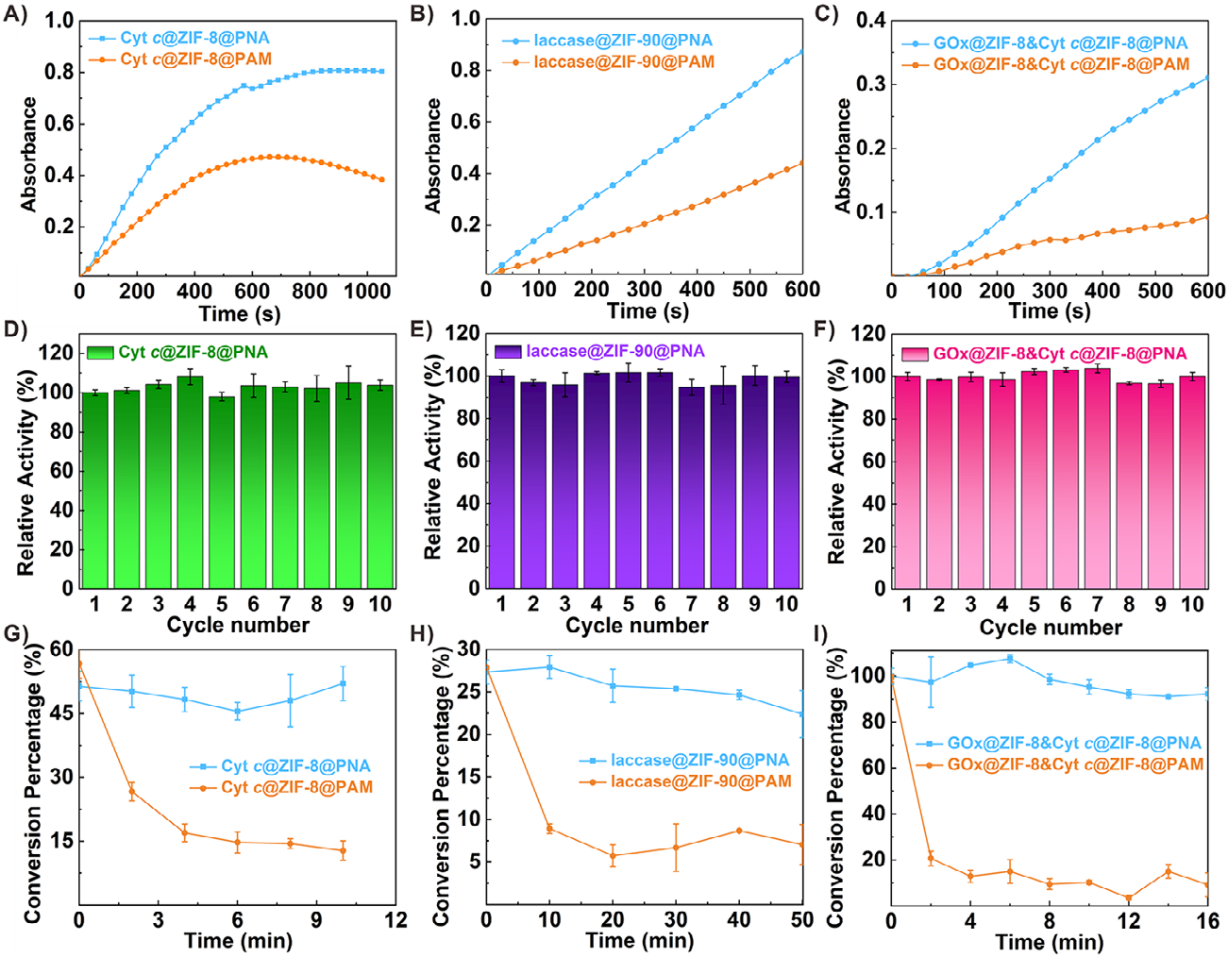
Verification of the universality. A,D,G) Comparison of catalytic activity, the regeneration property, and continuous H_2_O_2_ conversion performance between Cyt *c*@ZIF‐8@PNA and Cyt *c*@ZIF‐8@PAM. B,E,H) Comparison of catalytic activity, the regeneration property, and continuous reactive blue 19 conversion performance between laccase@ZIF‐90@PNA and laccase@ZIF‐90@PAM. C,F,I) Comparison of cascade catalytic activity, the regeneration property, and continuous glucose conversion performance between GOx@ZIF‐8&Cyt *c*@ZIF‐8@PNA and GOx@ZIF‐8&Cyt *c*@ZIF‐8@PAM.

To verify the broad applicability of this active pump‐in/out strategy in bulk hydrogel‐based biosystems, its capacity to facilitate other biocatalytic reactions within the hierarchical MOF‐doped PNA was further explored. Based on the enzyme characteristics, MOFs and immobilization methods were initially matched according to the literature to prepare cytochrome *c* (Cyt *c*)@ZIF‐8 and laccase@ZIF‐90, respectively (Figures S28 and S29, Supporting Information).^[^ ^41^, ^42^ ^]^ As a benchmarking experiment, we compared the in situ encapsulation approach with the conventional enzyme immobilization method‐specifically, enzyme adsorption onto ZIFs. The results showed that this method is significantly limited by low enzyme loading capacity owing to restricted pore accessibility and weak binding interactions (Figures S30 and S31, Supporting Information). Specifically, the loading capacities for GOx, Cyt *c*, and laccase were only 20%, 11%, and 13% of those achieved by the in situ method, respectively (Figure S32, Supporting Information). This leads to poor enzymatic performance to generate robust and representative activity data (Figure S33–S36, Supporting Information). So, the in situ method was applied for the further experiments. They were embedded in PNA and PAM using the same procedure, and their catalytic activities were then tested. As expected, compared to its passive‐infiltrated counterpart, the activity of the contracted Cyt *c*@ZIF‐8@PNA exhibited a dramatic improvement of 171% (**Figure** **4**A). The value for laccase@ZIF‐90@PNA was 117% (Figure 4B). Additionally, because two or more enzymes are often required for advanced functions and practical applications, GOx@ZIF‐8 and Cyt *c*@ZIF‐8 were co‐doped to perform the cascade reaction. The activity test suggested an improved reaction rate (167.8%) by introducing active mass transfer (Figure 4C). These results validate that the interior dynamic water stream generated by the expansion of PNA not only accelerates the enzyme response to substrates in the external environment but also enhances mass transfer between different reactors within the bulk hydrogel. Moreover, all the above biosystems were easily regenerated (Figure 4D–F) and exhibited stable substrate conversion efficiencies, even after multiple reuse cycles or extended periods of time (Figure 4G–I; Figures S37–S39, Supporting Information).

### 2.5 The Application of Enzyme@MOFs@PNA in Biosensing

**Figure 5:**
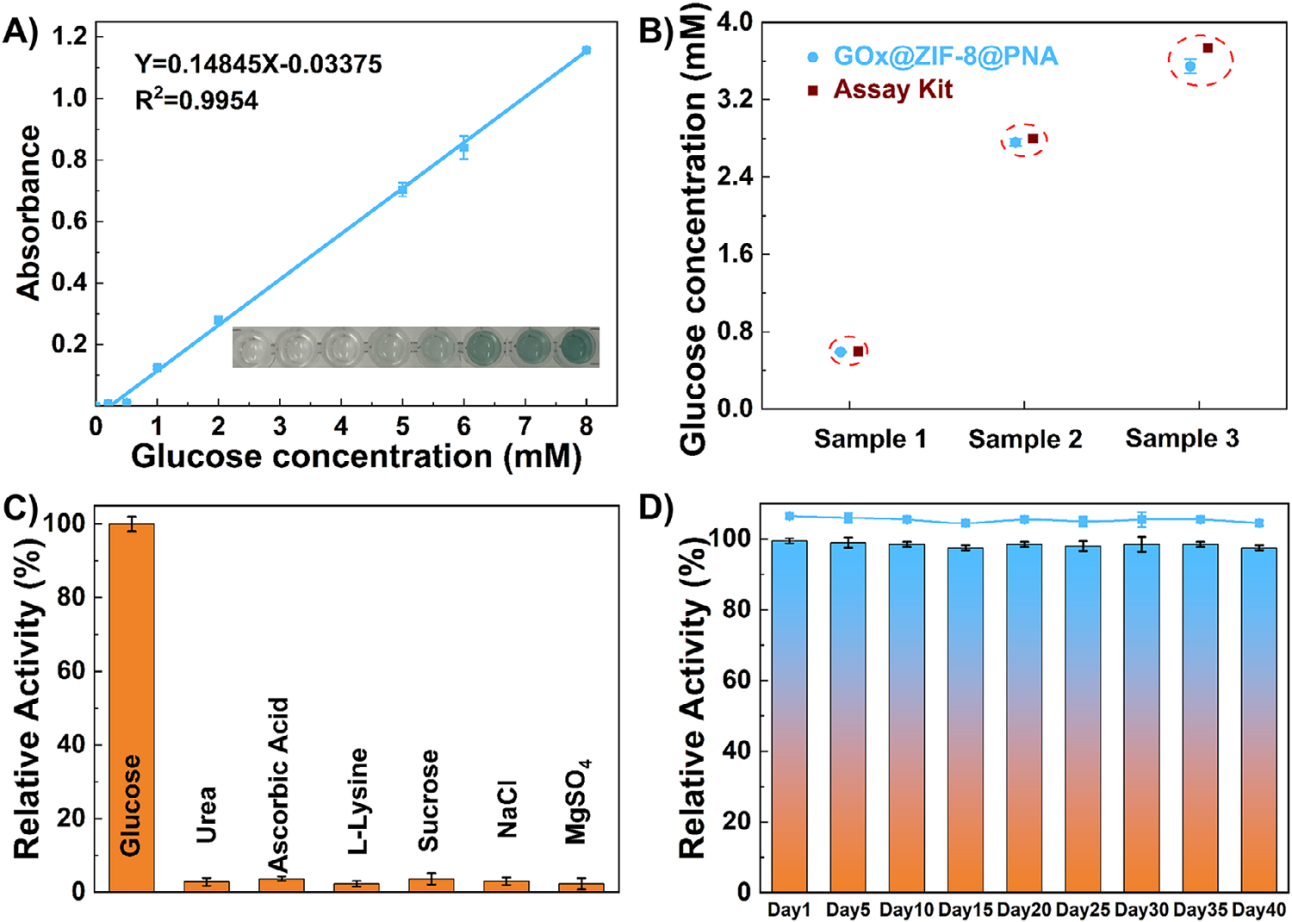
Glucose detection based on the biological cascade method. A) A linear calibration curve for glucose determined at 418 nm (Inset: the corresponding colorimetric photograph). B) The detection results of artificial perspiration (sample 1), pig serum (sample 2), and fetal bovine serum (sample 3) using GOx@ZIF‐8@PNA and their comparison with commercial detection results. C) Selectivity of GOx@ZIF‐8@PNA toward glucose. D) Long‐term detection stability of GOx@ZIF‐8@PNA.

Based on the construction and advantages of the above enzyme immobilization system, we investigated the application of the model system GOx@ZIF‐8@PNA in glucose sensing. The results showed that the biosensor exhibited a good linear relationship within the concentration range of 0–8 mM (**Figure** **5**A), with a detection limit as low as 230 µm (LOD = 3.3 δ/S). The performance of the biosensor was further evaluated in various samples, including artificial sweat, pig serum, and fetal bovine serum, yielding results highly consistent with those obtained using commercial glucose detection kits (Figure 5B). Moreover, due to the high selectivity of enzymatic catalysis, the biocascade‐driven sensing pathway endowed this portable biosensor with ultrahigh specificity for glucose, as evidenced by the negligible color change observed upon adding 6 other biomolecules at concentrations 10 times higher than that of glucose (Figure 5C). Additionally, the sensing stability of GOx@ZIF‐8@PNA could be almost fully retained after storage at room temperature for 40 days (Figure 5D).

## 3 Conclusion

This study presents a general and scalable design principle for fabricating active, metabolism‐capable MOFs‐doped hydrogel for enzyme immobilization. This approach overcomes the challenges of poor activity and recyclability of hydrogel‐based biosystems, which are often limited by restricted mass transfer and irregular accumulation or leakage in bulk structures. The smart hydrogels and MOFs work together to protect enzymes from denaturation and enable the regeneration of biosystems for repeated use. Similar to the cardiac pumping process, the thermally triggered rapid contraction/expansion of the PNA hydrogel creates a cyclic dynamic water pump‐in/out, facilitating the efficient intake of substances and expulsion of endogenous products. The inclusion of MOFs provides an interception effect, preventing enzyme leakage. These advancements offer new insights into enzyme behavior in both in vivo and in vitro 3D soft systems and expand the possibilities for developing intelligent wearable sensors, microdevices, and even artificial organelles.

## Conflict of Interest

The authors declare no conflict of interest.

## Author Contributions

Y.X. and S.H. contributed equally to this work. Y.Z. was responsible for methodology and data curation, while S.W. contributed to data curation, formal analysis, software, and the original draft. Y.L. assisted with data curation, and L.C. contributed to software development. X.G. provided resources and secured funding, whereas Y.Zh. supervised the work. M.L. contributed to conceptualization, validation, data curation, and review and editing of the manuscript. T.W. acquired funding, supervised the project, managed administration, and participated in review and editing

## Supporting information



Supporting Information

## Data Availability

The data that support the findings of this study are available from the corresponding author upon reasonable request.
