# Supplementary material for: Smart Hydrogel Doped by Metal–Organic Frameworks for Renewable Self‐Pumping Enzymatic Reactors
Source: Adv Sci (Weinh). 2025 Nov 29;13(9):e07886. doi: 10.1002/advs.202507886 (PMC12904019; doi:10.1002/advs.202507886)
Supplement: Supplementary file 1 — Supporting Information [file ADVS-13-e07886-s001.docx]

Supporting Information

Smart Hydrogel Doped by Metal-Organic Frameworks for Renewable Self-Pumping Enzymatic Reactors

Yuxi Zhu ^+^, Shenghao Wang^+^, Yueyuan Luo, Linbo Cao, Xiuxiu Guo, Yue Zhang, Mingmin Li* and Tie Wang*

^+^ Y. X. and S. H. contributed equally to this work.

S1. Details on experimental method

Materials

Except for special description, all materials including solvents throughout the experiments were purchased from commercial sources and used as received without further purification. Glucose oxidase (GOx), DNS reagent and Bradford reagent were purchased from Solarbio (Beijing, China). HEPES, 2-methylimidazole (2-MIM), horseradish peroxidase (HRP), *N*-isopropylacrylamide (NIPAM), methylene blue and reactive blue 19 were obtained from Macklin (Shanghai, China). 2-Hydroxy-1-(4-(2-hydroxyethoxy)phenyl)-2-methylpropan-1-one was purchased from Bidepharm (Shanghai, China). 2,2'-azinobis(3-ethylbenzothiazoline-6-sulfonic acid ammonium salt) (ABTS), dimethyl sulfoxide (DMSO), acetate, zinc acetate and polyvinyl pyrrolidone (PVP) were purchased from Aladdin (Shanghai, China). Acrylamide was purchased from Innochem (Beijing, China). *N*,*N*,*N*',*N*'-tetramethylethylenediamine (TEMED) was purchased from Beyotime (Shanghai, China). Sulfuric acid, NaOH, H_2_O_2_ and acetone was purchased from Fengchuan (Tianjin, China). Potassium bromide was purchased from Damao (Tianjin, China). *N*,*N*'-Methylenebisacrylamide was purchased from Rhawn (Shanghai, China). MeOH and *N*,*N*-Dimethylformamide (DMF) was purchased from Bocheng (Shandong, China). Zinc nitrate was purchased from Kemiou (Tianjin, China). Cytochrome *c* (Cyt *c*) from pig was purchased from MeilunBio (Dalian, China). Laccase was purchased from Yuanye (Shanghai, China). 2-imidazolylcarboxaldehyde (ICA) was purchased from Shaoyuan (Shanghai, China). Xylenol orange was purchased from TCL (Shanghai, China). D-Sorbitol and ammonium iron (II) sulfate hexahydrate was purchased from HEOWNS (Tianjin, China).

**Methods**

*Powder X-ray diffraction (PXRD)*: PXRD pattern of all materials were collected at ambient temperature with a Rigaku Ultima Iv diffractometer operated at 40 kV and 40 mA using Cu Kα (λ = 1.5418Å) radiation, with a scan speed of 1 sec/step, a step size of 0.02° in 2θ, and a 2θ range of 5° to 40°.

*Ultrahigh-resolution scanning electron microscope (HESEM)*: As-prepared hydrogel samples were immersed in liquid nitrogen for instant freezing. Then they were freeze-dried for 24 h with a freeze dryer and observed under the Ultrahigh-Resolution Scanning Electron Microscope of FEI Compact in America with an accelerating voltage of 15 kV.

*Transmission electron microscope (TEM)*: JEM-1400Flash at an accelerating voltage of 120 kV was applied to keep track of TEM images. For samples preparation, a drop of ethanol suspension containing the samples were added on a carbon grid and dried at room temperature.

*Fourier transform infrared (FT-IR) spectra*: FT-IR spectrums of all materials were obtained on the Frontier Mid-IR FTIR spectrometer.

*Rheological tests*: The mechanical properties of the prepared fresh gel samples were tested by modular intelligent advanced rheometer (MCR302). The oscillation strain scanning test was carried out in the range of 0.01%-100%, and the oscillation time scanning test was carried out at a fixed frequency of 1 Hz.

*Mechanical property tests of hydrogels*: The premixed solution was completely gelled into a cylindrical shape with a height of 0.5 cm and a diameter of 1.7 cm. An in-situ thermoelectric cooling stage was used to stretch the samples at a speed of 100 μm·s^-1^.

*Fluorescence test*: The fluorescence intensity detection was applied on a fluorescence spectrophotometer (RF-6000) with an excitation wavelength of 600 nm, and the emission spectrum was collected during 650-800 nm. The AniView100 (BLT, Guangzhou, China) was used to observe the Methylene blue distribution inside the hydrogel.

*UV-Vis spectrum*: UV-1900i ultraviolet-visible spectrophotometer of Shimadzu Company in Japan was used to record the catalytic process. The absorbance value was employed with SpectraMax ID3 microplate reader.

**Material synthesis procedures**

*Preparation of hydrogels*: NIPAM was prepared according to the literature reported with a slight modification as follows.^[1]^ In brief, 44 mg NIPAM was dissolved in the solution of 2-Hydroxy-1-(4-(2-hydroxyethoxy)phenyl)-2-methylpropan-1-one (0.25 mL, 15.7 mM) and *N*,*N*'-Methylenebisacrylamide (0.25 mL, 11.42 mM). Then the mixture was sonicated for 5 min until completely dissolved. Then 14.4 μL TEMED was added as a reaction accelerator. The reaction mixture was deoxygenated under a N_2_ atmosphere for 10 min and finally cured by ultraviolet light for 5 min. The same protocol was conducted for the preparation of PNIPAM-PAM (PNA), where 43 mg NIPAM and 1.1 mg AM was used instead of pure NIPAM. Also, 44 mg AM was applied to prepare PAM.

*Preparation of enzymes@ZIF-8*: GOx@ZIF-8 was synthesized via de-novo method as follows: 0.4105 g of 2-MIM was dissolved into 4 mL of DI water with the addition of 4.4 mg GOx. Then Zn(NO_3_)_2_·6H_2_O (0.4 mL, 309.25 mM) was added to the above solution. The reaction mixture was kept still for 1 h at room temperature. The product was centrifuged (8000 rpm, 3 min) and washed with deionized water for 3 times. Finally, the obtained powder was dried by an air pump and stored at 4 ℃ for further use. For the preparation of Cyt *c*@ZIF-8, the same protocol was applied where Cyt *c* (4.4 mg) was added instead of GOx. Pure ZIF-8 was prepared without enzyme through the same steps. As control experiment, the adsorption of GOx or Cyt *c* onto the ZIF-8 surface was investigated. A solution containing 1 mg/mL enzyme was incubated with 6 mg/mL of ZIF-8 in a total volume of 4.4 mL. At predetermined time intervals, the mixture was centrifuged, and 100 μL aliquots of the supernatant were withdrawn for spectrophotometric quantification of protein concentration. Adsorption kinetics and final loading capacities were determined by monitoring the change in protein concentration over time.

*Preparation of laccase@ZIF-90*: Zinc nitrate (371.3 mg) was added into DI water (3 mL) as a triggered solvent. Then, this solution was added to DI water (25 mL), in which ICA (480.0 mg), PVP (50.0 mg) and laccase (5 mg) was dissolved. The reaction mixture was stirred for 10 min at room temperature. The product was collected by centrifugation at 5500 rpm for 10 min, washed three times with DI water and dried at room temperature. Pure ZIF-90 was prepared without enzyme by the above steps. The adsorption of laccase onto the ZIF-9 surface was carried out using the same procedure as for GOx and Cyt *c*, with a laccase concentration of 0.18 mg/mL and a ZIF-90 concentration of 2.2 mg/mL.

*Preparation of GOx@PNA, enzymes@MOFs@PNA and enzymes@MOFs@PAM*: For the preparation of GOx@ZIF-8@PNA, 43 mg NIPAM and 1 mg AM was dissolved in 2-Hydroxy-1-(4-(2-hydroxyethoxy)phenyl)-2-methylpropan-1-one (0.25 mL, 15.7 mM), *N*,*N*'-Methylenebisacrylamide (0.25 mL, 11.42 mM) and sonicated for 5 min to make them completely dissolved. Then 14.4 μL TEMED was added as a reaction accelerator. The above mixture was deoxygenated under a N_2_ atmosphere for 10 min, after which 5.2 mg GOx@ZIF-8 was added, and finally it was cured by ultraviolet light for 5 min. Similarly, 2 mg GOx was used instead of GOx@ZIF-8 to prepare GOx@PNA. The same protocol was applied to prepare Cyt *c*@ZIF-8@PNA, laccase@ZIF-90@PNA and GOx@ZIF-8&Cyt *c*@ZIF-8@PNA, the UV irradiation time was extended to 10 min or 15 min. When 44 mg AM is used instead of 43 mg NIPAM and 1 mg AM, and other preparation processes remain unchanged, the corresponding enzymes@MOFs@PAM can be prepared.

*Adsorption of methylene blue by PNA/PAM*: PAM or shrunken PNA was soaked in 0.03 mg/mL methylene blue aqueous solution, and the fluorescence images and UV-vis light absorption intensity of hydrogels were measured at certain time intervals.

*Release of methylene blue by PNA/PAM*: After the hydrogels were completely saturated by dye, the surface adsorbed methylene blue was firstly washed by DI water. The obtained hydrogels were then soaked in water of 45 ℃ for 2 min. The supernatant was collected for UV-vis spectrum test. Meanwhile, the hydrogels were incubated with water of 25 ℃ to perform expansion and water pump-in process. All above operation was recorded as one cycle, which was continuously repeated for several times to realize complete pump-out of methylene blue.

*Enzymatic activity of GOx@ZIF-8@PNA*: The as-prepared GOx@ZIF-8@PNA were non-heated or thermal treated to shrink to different sizes (i. e., 100%, 80%, 67% and 41% to their original volume). Then, their activities were measured respectively. Typically, HEPES buffer (845 μL, 50 mM, pH=7) was added into GOx@ZIF-8@PNA, then ABTS (100 μL, 10 mM), HRP (5 μL, 1 mg/mL) and glucose solution (50 μL, 25 mM) were successively added, and the systems were continuously monitored by SpectraMax ID3 microplate reader at 418 nm.

*Tolerance evaluation in perturbation environments*: GOx, GOx@ZIF-8, GOx@PNA and GOx@ZIF-8@PNA were incubated in different conditions (including acetone for 15 min, DMSO for 5 min, 60 ℃ for 30 min and MeOH for 30 min) to evaluate their tolerance. After above treatments, their activities were continuously monitored by SpectraMax ID3 microplate reader at 418 nm.

*The regeneration of enzymes@MOFs@PNA*: After completing one catalytic cycle, the bio-hydrogels were soaked in heat water of 45 ℃ to pump out the interior products and unreacted substrates. Then it was immersed in pure cold water for expansion. This cyclic procedure was repeated several times until the accumulation was completely excreted to the outside of the body. The pumped liquid was collected to detect the leakage of enzymes during this process using the Bradford method.

*GOx catalysis in continuous-flow reaction system*: Typically, the column reactor (with an inner diameter of 1 cm) was packed with a piece of contracted GOx@ZIF-8@PNA and filled with degreasing cotton at the outlet to prevent the hydrogel from being washed away. The above reactor was fixed on a continuous conversion device. Then 1 mM glucose solution and O_2_ were simultaneously pumped into the column reactor at a specific flow rate of 1.2 mL/min. At certain time intervals, the column reactor was heat/ice treated repeatedly at 45 °C/0 °C to regenerate the catalytic system. For control experiment of GOx@ZIF-8@PAM, the column temperature was kept constant during the process. The outflow was collected at intervals for residual glucose concentration analysis through 3,5-dinitrosalicylic acid (DNS) method. In brief, 50 μL of outlet solution was handed over and mixed with 50 μL DNS solution. The mixtures were kept at 100 ℃ for 5 min, after cooling to room temperature, they were diluted five times with 400 μL DI water. The absorbance at 560 nm was measured to monitor the concentrations of glucose, which was applied to determine the conversion percentage.

*Enzymatic activity of Cyt c@ZIF-8@PNA*: The activity of Cyt *c* based catalytic system was determined by a colorimetric method based on ABTS oxidation. Typically, Contracted Cyt *c*@ZIF-8@PNA was dispersed into HEPES buffer (850 μL, 50 mM, pH=7). Then ABTS (100 μL, 10 mM) and H_2_O_2_ (50 μL, 20 mM) were successively added, and the system was continuously monitored by SpectraMax ID3 microplate reader at 418 nm. Also, Cyt *c*@ZIF-8@PAM in swollen state was set as control group.

*Enzymatic activity of laccase@ZIF-90@PNA*: The activity of laccase based catalytic system was determined by colorimetry based on ABTS oxidation. Typically, contracted laccase@ZIF-90@PNA was dispersed into acetic acid buffer (900 μL, 0.2 M, PH=5.0). Then ABTS (100 μL, 10 mM) was successively added, and the system was continuously monitored by SpectraMax ID3 microplate reader at 418 nm.

*Enzymatic activity of GOx@ZIF-8&Cyt c@ZIF-8@PNA*: The activity test method of GOx@ZIF-8&Cyt *c*@ZIF-8@PNA was similar to that of GOx@ZIF-8@PNA except that HRP was not necessary.

*Laccase catalyzes the decomposition of reactive blue 19*: The reactive blue 19 (0.2 mg/mL, 1 mL) was added to the laccase solution (2 mg/mL, 1 mL) to initiate the degradation reaction, and the absorbance of the solution at 590 nm was monitored at different time intervals.

*Cyt c catalysis in continuous-flow reaction system*: This reaction system was similar to that of GOx catalysis in continuous-flow reaction system, excepting that 20 mM H_2_O_2_ was pumped in instead of glucose solution and O_2_. The outlet solution was collected for H_2_O_2_ quantification. In detail, the solution was firstly diluted by 20-fold, from which 10 μL was taken out and added to 190 μL of FOX reagent.^[2]^ The mixture was incubated for 30 min at room temperature for absorbance value test at 560 nm. The conversion rate was calculated by calculating the corresponding residual H_2_O_2_ concentration through the linear curve.

*Laccase catalysis in continuous-flow reaction system*: A device similar to above Cyt *c* catalyst flow reaction system was used, excepting that hydrogen peroxide solution was replaced by 0.02 mg/mL reactive blue 19 solution. The absorbance of outlet solution at 590 nm was monitored at different time intervals, and the corresponding reactive blue 19 concentration was determined by the linear curve to calculate the conversion rate.

*Continuous flow cascade catalytic system:* This reaction system was identical to that of GOx catalysis in continuous-flow reaction system.

*Evaluation of Glucose Detection Selectivity*: The selectivity of the GOx@ZIF-8@PNA system for glucose was assessed in solutions containing either glucose (1.25 mM) or interfering substances (urea, ascorbic acid, L-lysine, sucrose, sodium chloride, magnesium sulfate, 12.5 mM). Specifically, HEPES buffer (845 μL, 50 mM, pH=7) was added to GOx@ZIF-8@PNA, followed by sequential addition of ABTS (100 μL, 10 mM), horseradish peroxidase (5 μL, 1 mg/mL), glucose solution (50 μL, 25 mM), or other interfering chemicals (50 μL, 250 mM). The system was continuously monitored at 418 nm using a UV-vis spectrophotometer.

**S2. Supplementary Figures**


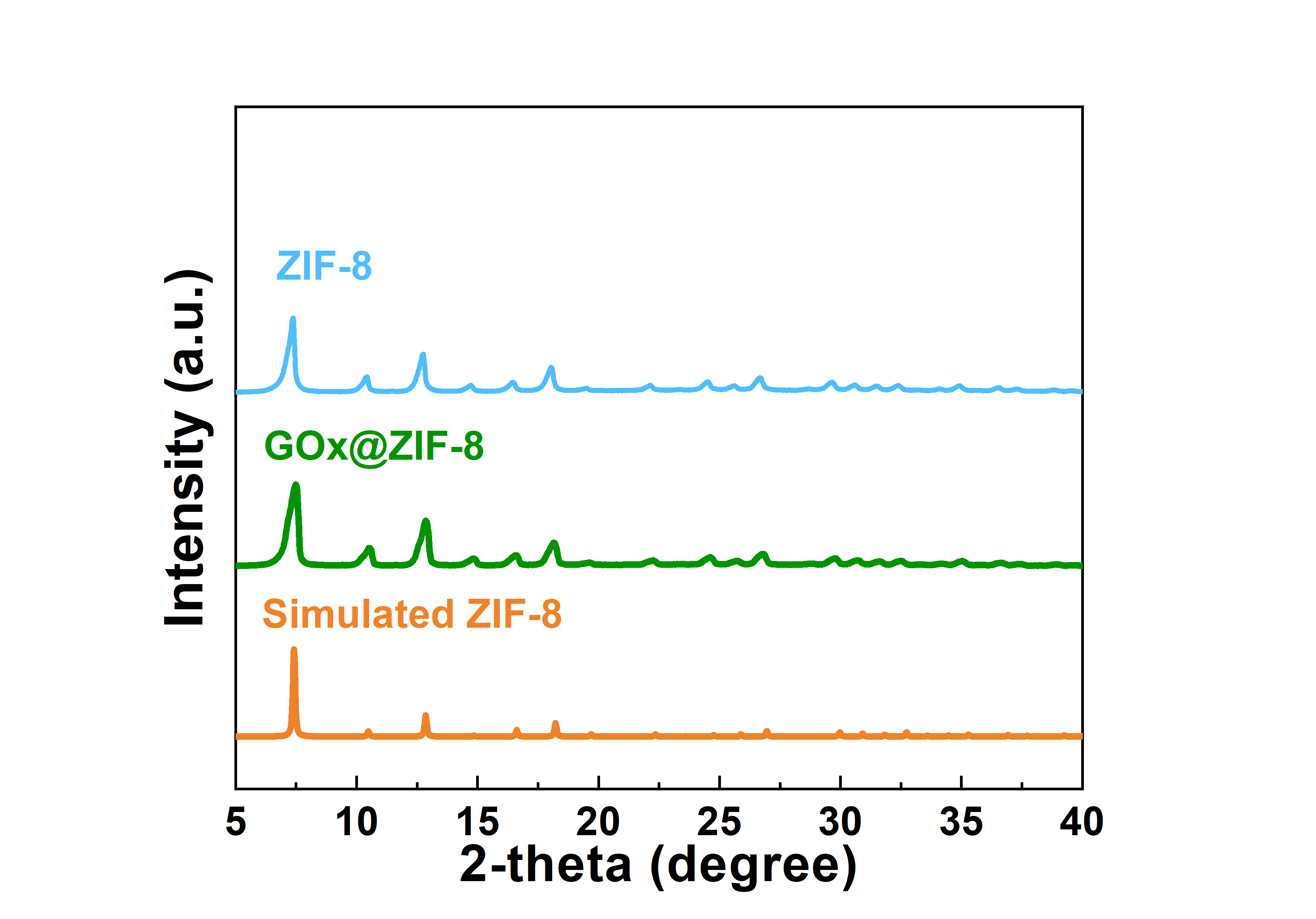


**Figure S1.** PXRD patterns of ZIF-8 and GOx@ZIF-8.


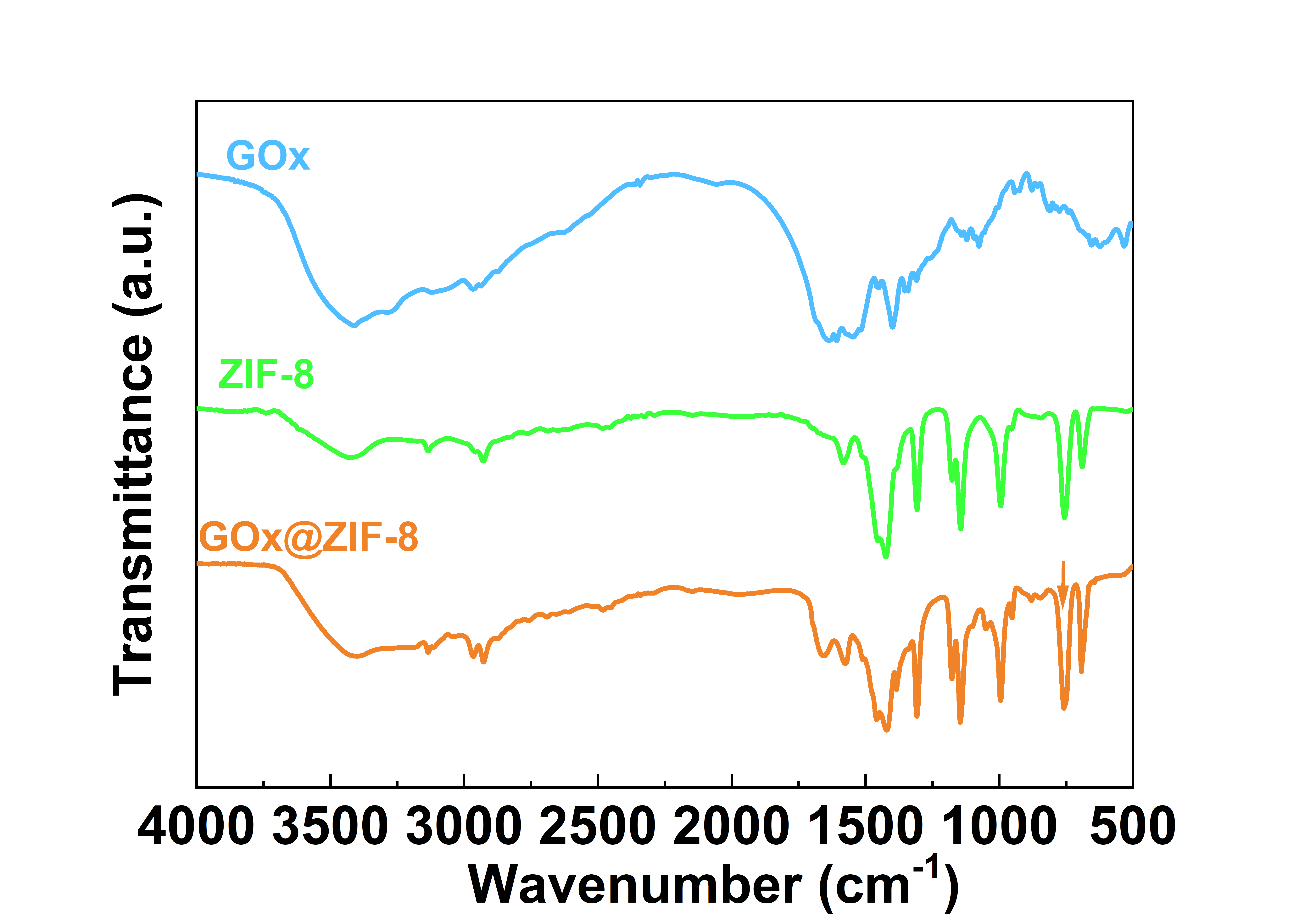


**Figure S2.** FT-IR spectra of GOx, ZIF-8 and GOx@ZIF-8.


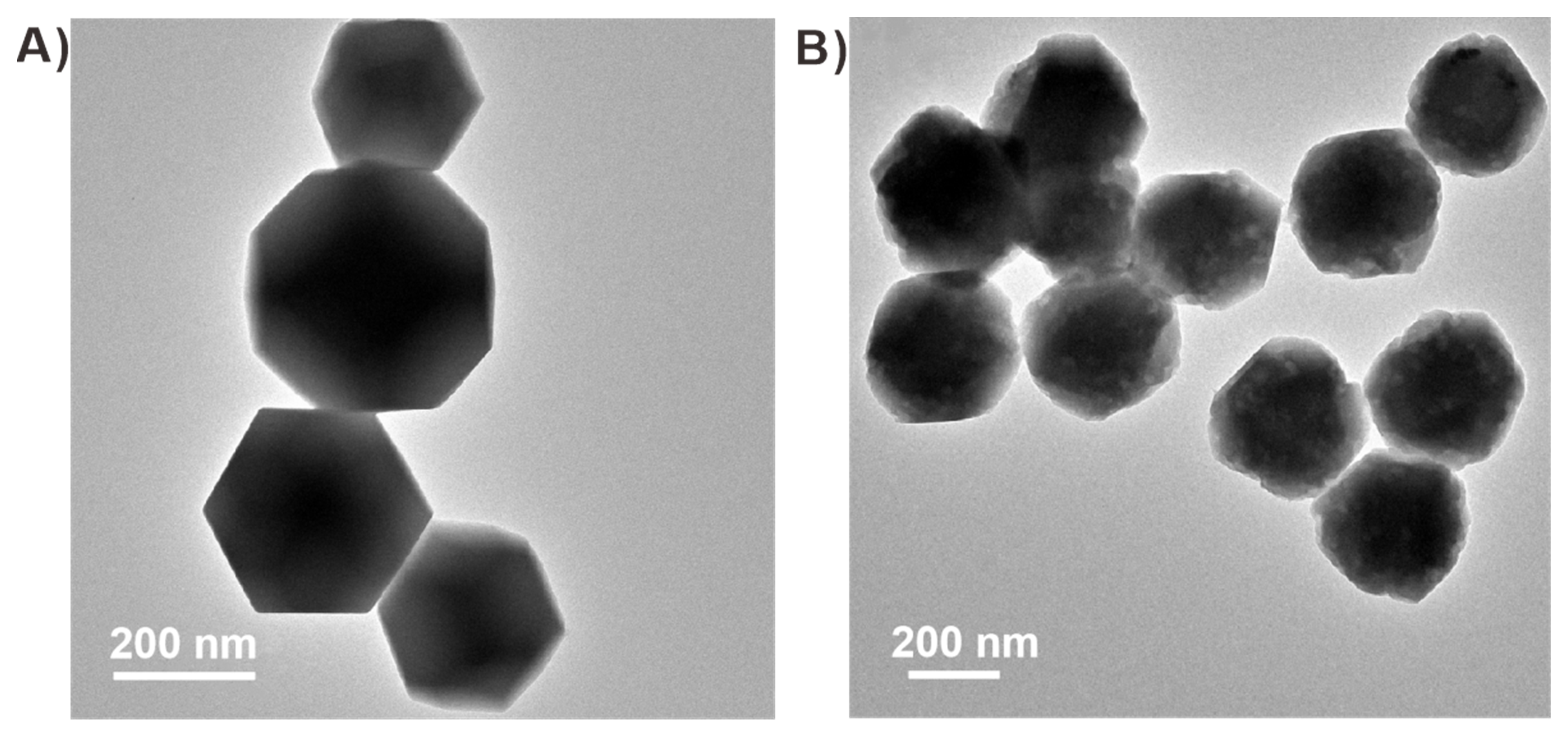


**Figure S3.** TEM images of **A)** ZIF-8 and **B)** GOx@ZIF-8.


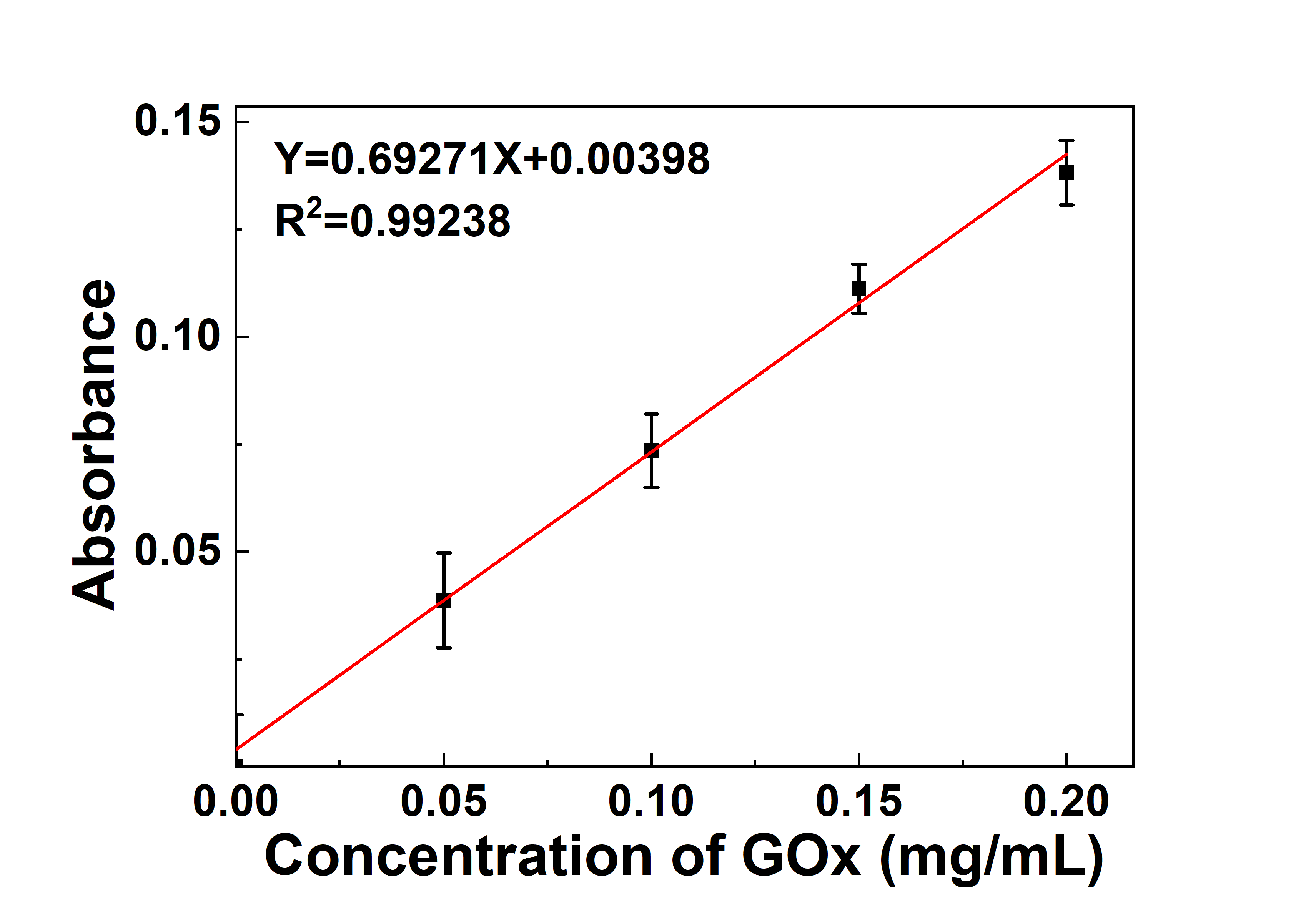


**Figure S4.** Standard curve of GOx based on Bradford method.


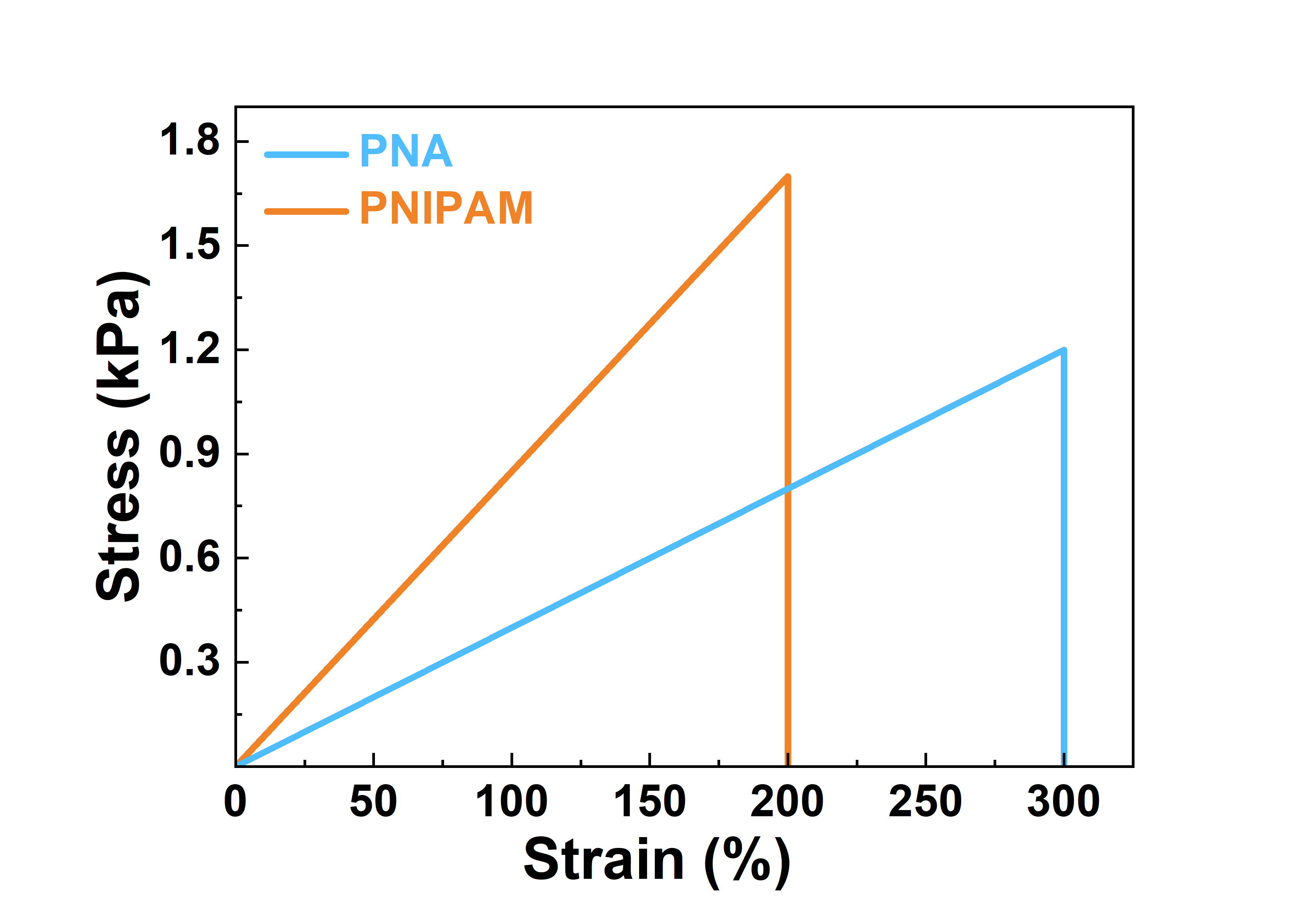


**Figure S5.** Stress-strain curves of PNIPAM and PNA gels.


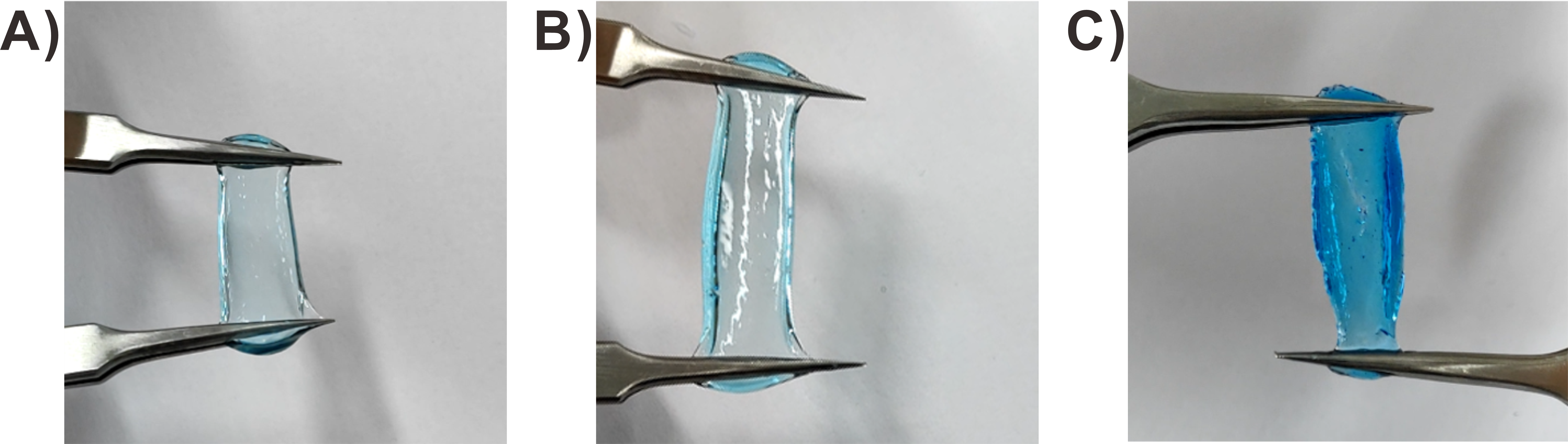


**Figure S6.** The tensile property comparison of **A)** PNIPAM, **B)** PAM and **C)** PNA.

**
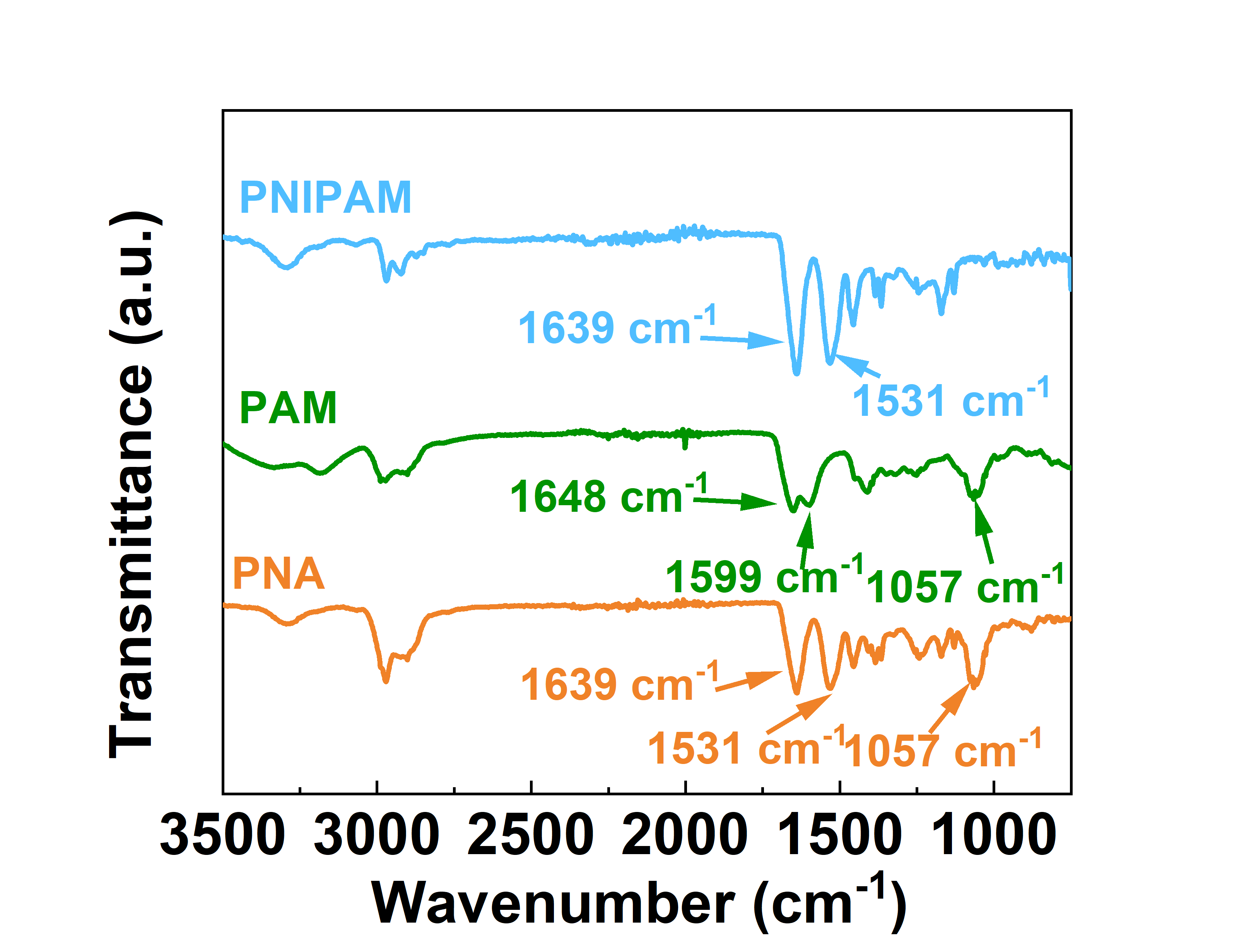
**

**Figure S7.** FT-IR spectra of PNIPAM, PAM and PNA gels.


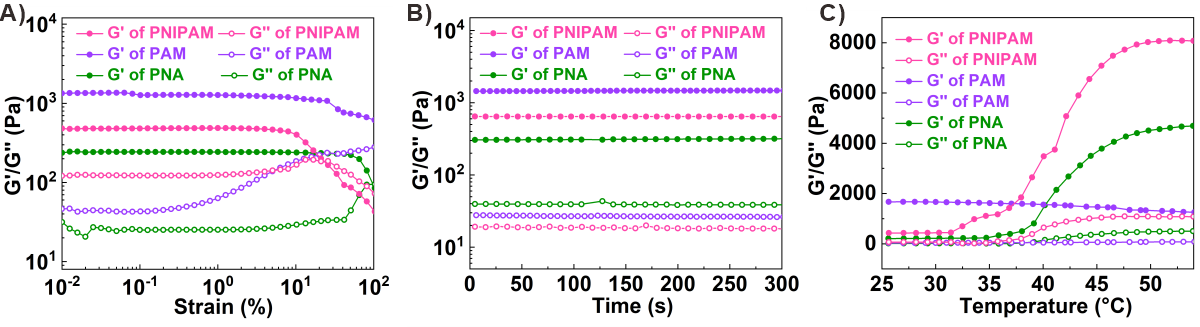


**Figure S8. A)** The oscillating strain scanning curves of three gels at a fixed frequency of 1 Hz. **B)** Oscillation time scanning curves of three gels at a fixed frequency of 1 Hz and 1% strain. **C)** Rheological behavior of three gels at different temperatures.


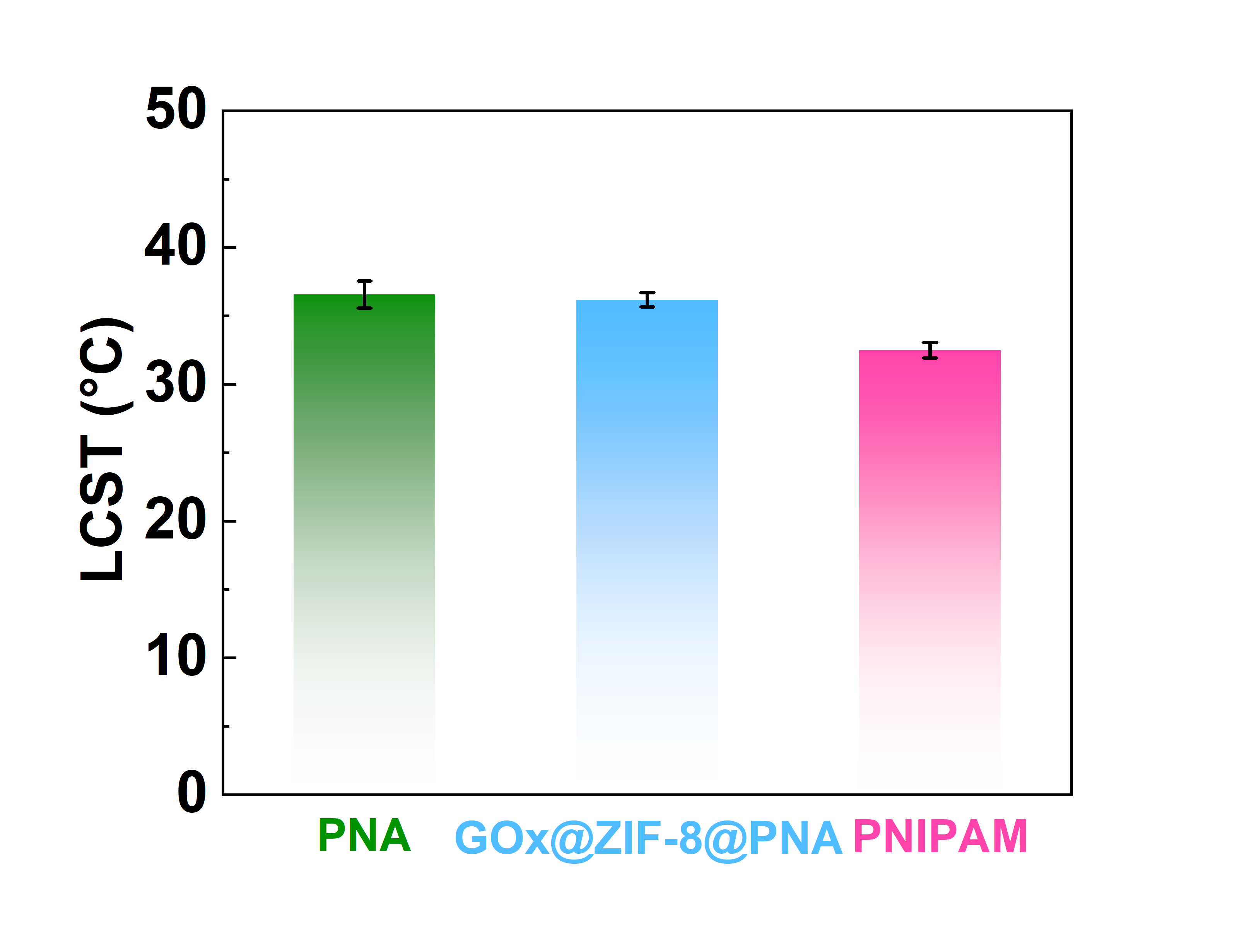


**Figure S9.** Comparison of lower critical solution temperature (LCST) of PNA, GOx@ZIF-8@PNA and PNIPAM.


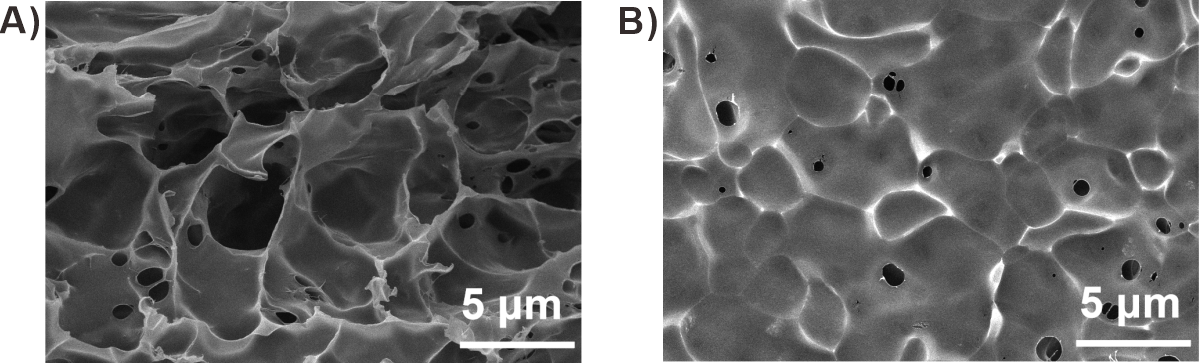


**Figure S10.** SEM images of partial region of PNA gel **A)** at 25 ℃ and **B)** at 45 ℃.


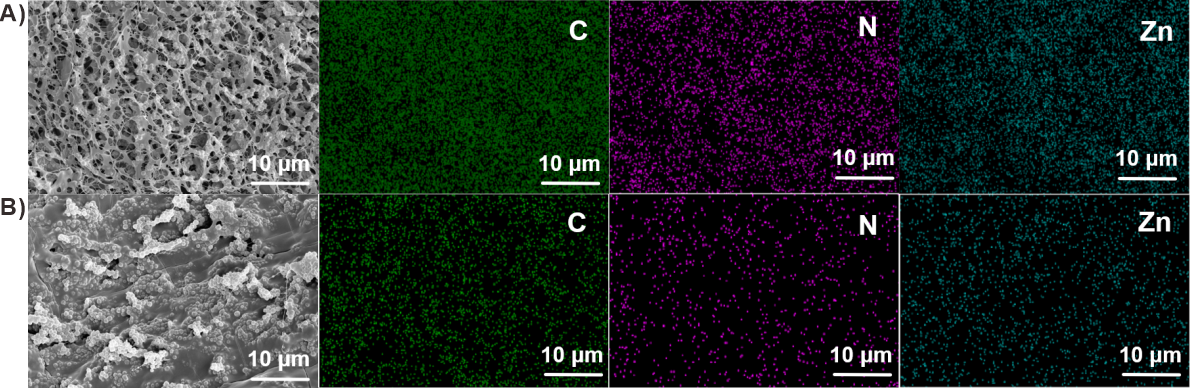


**Figure S11.** The HESEM images and element mapping of GOx@ZIF-8@PNA at **A)** expansive and **B)** contracted states.


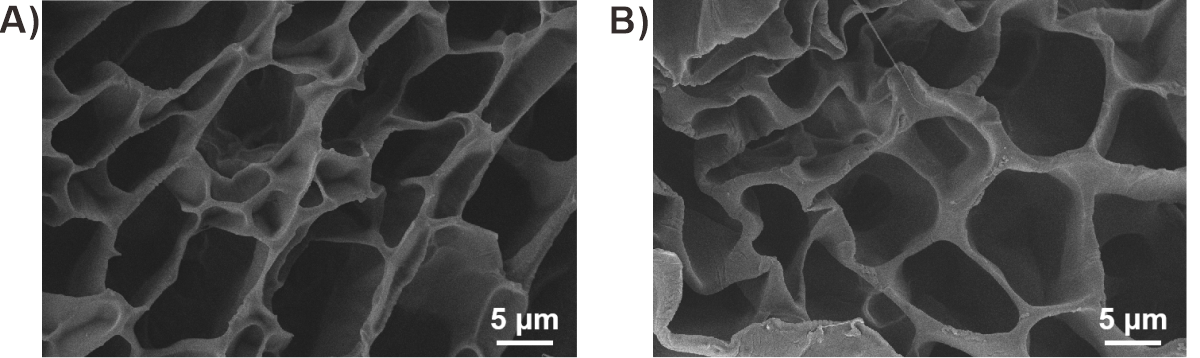


**Figure S12.** SEM images of partial region of PAM gel **A)** at 25 ℃ and **B)** at 45 ℃.


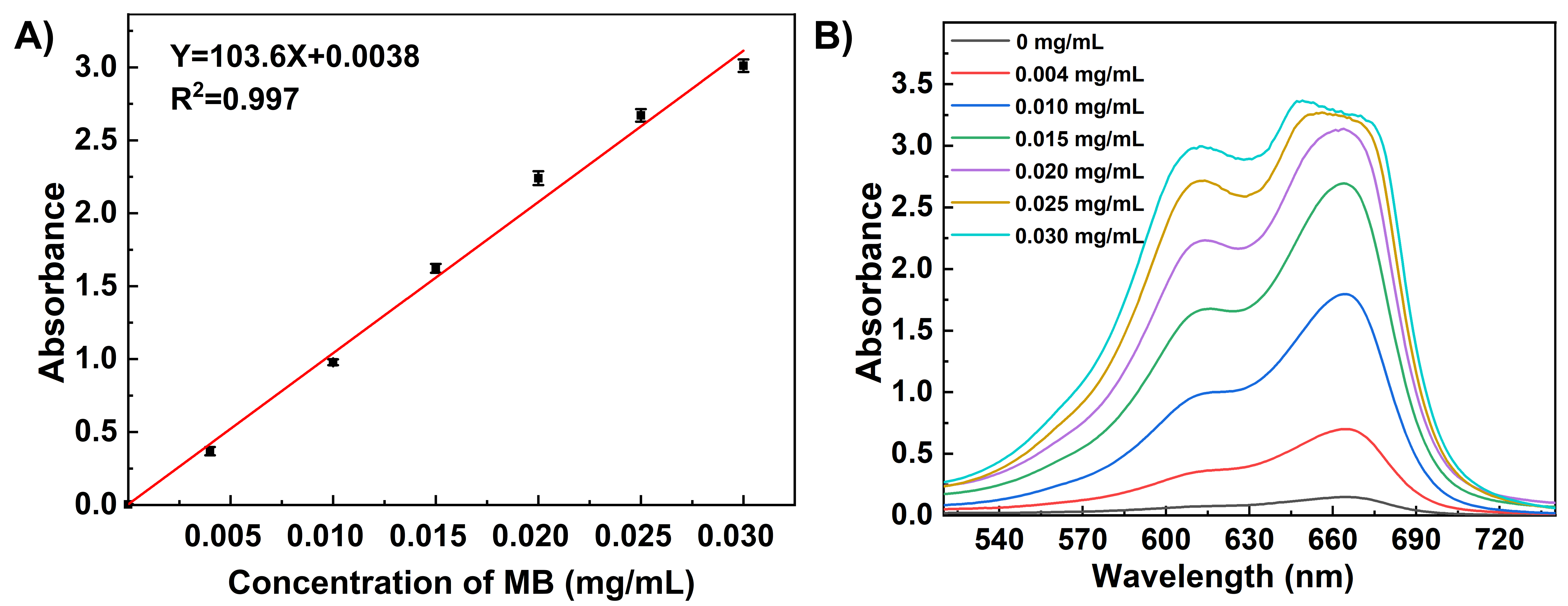


**Figure S13.** **A)** Standard curve of MB aqueous solution. **B)** UV-Vis absorption spectra of MB aqueous solution with different concentrations.


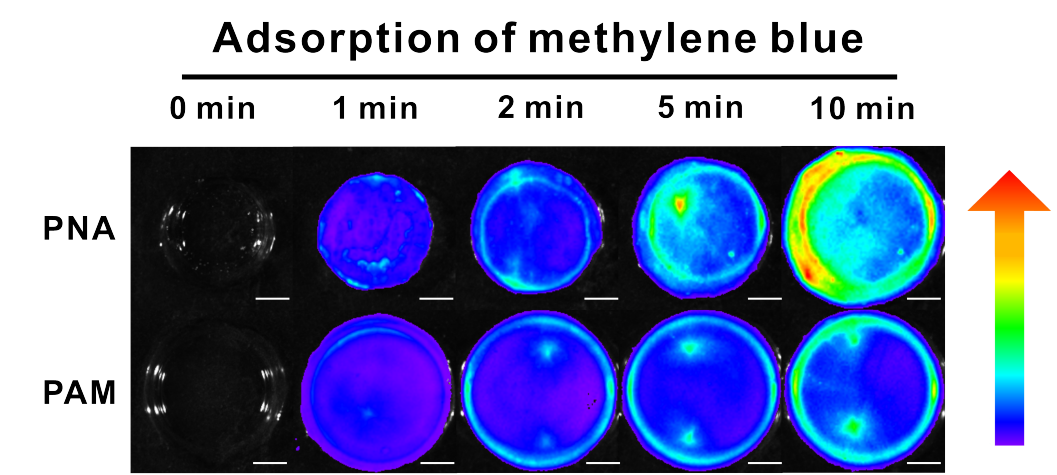


**Figure S14.** Fluorescence images of PNA and PAM at certain time intervals during the adsorption of methylene blue (scale bars: 1 cm).


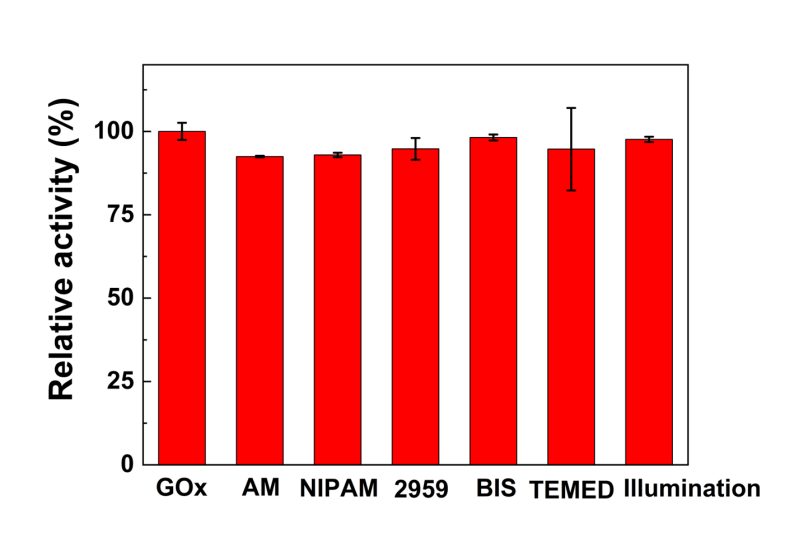


**Figure S15.** The influence of various raw materials as well as illumination condition used in the preparation of GOx@ZIF-8@PNA toward the catalytic activity of GOx.


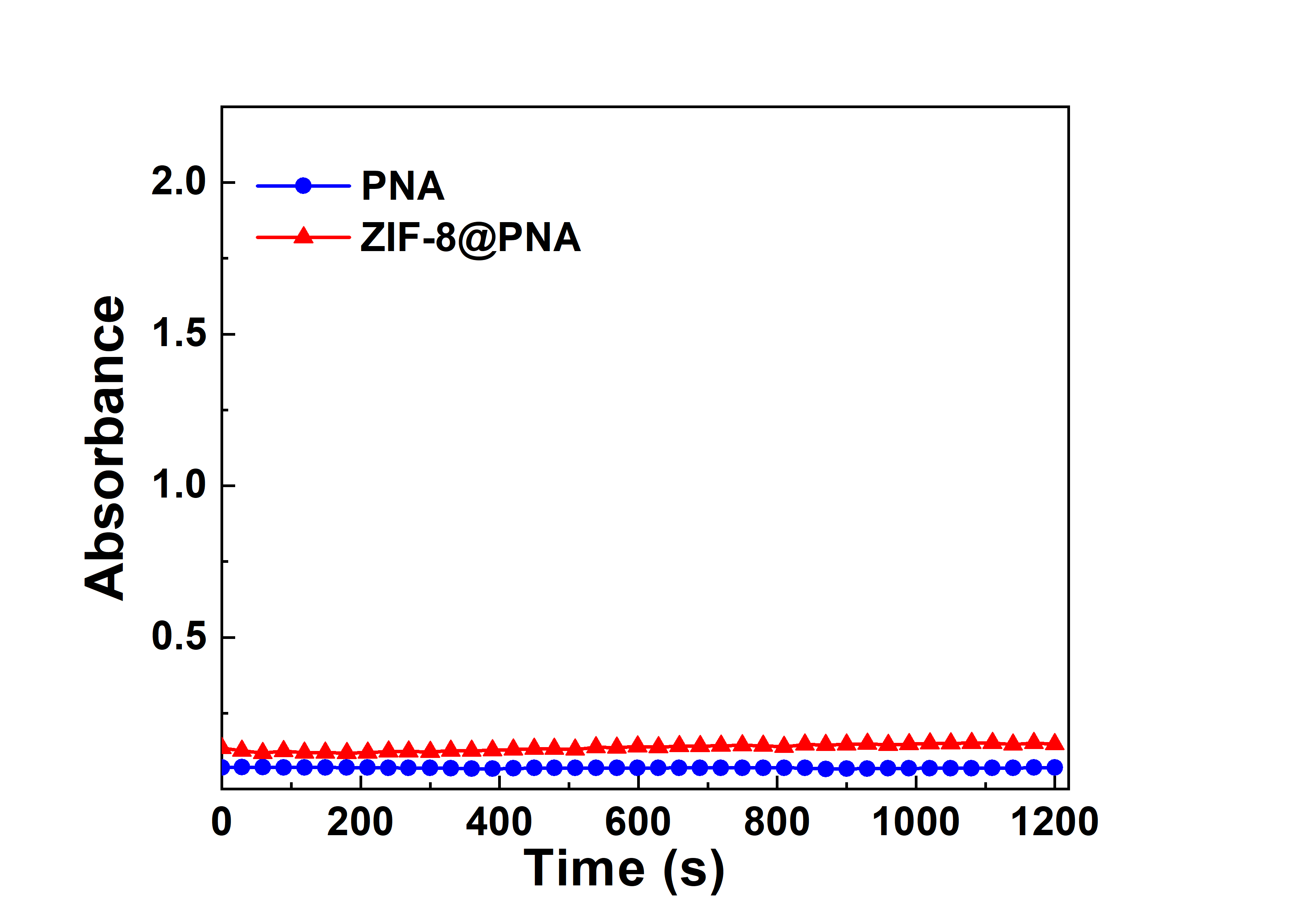


**Figure S16.** The catalytic activity of pure ZIF-8 and PNA without GOx.


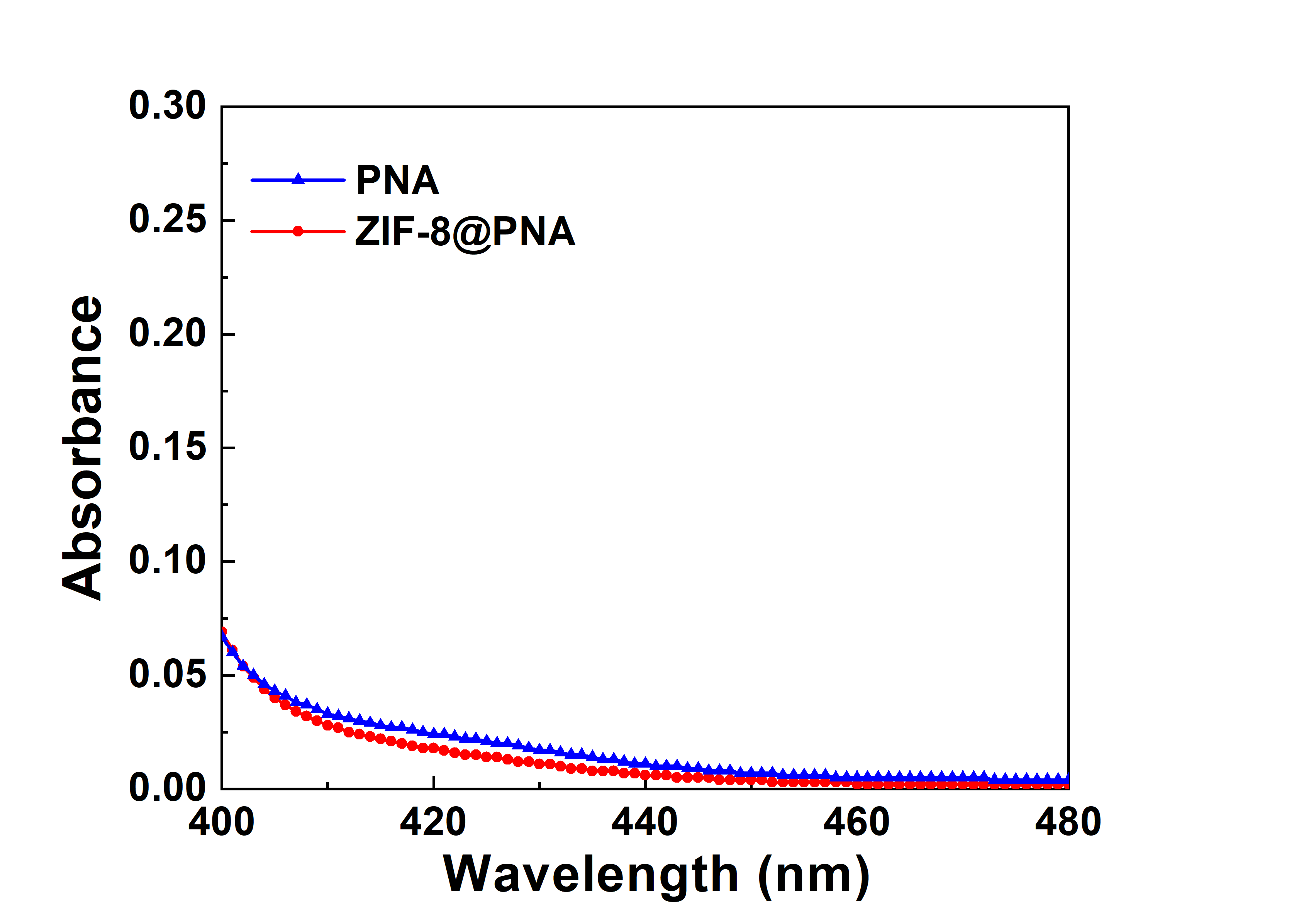


**Figure S17.** UV-Vis spectra of pure ZIF-8 and PNA without GOx after incubation with the reactive substrate for 12 min.


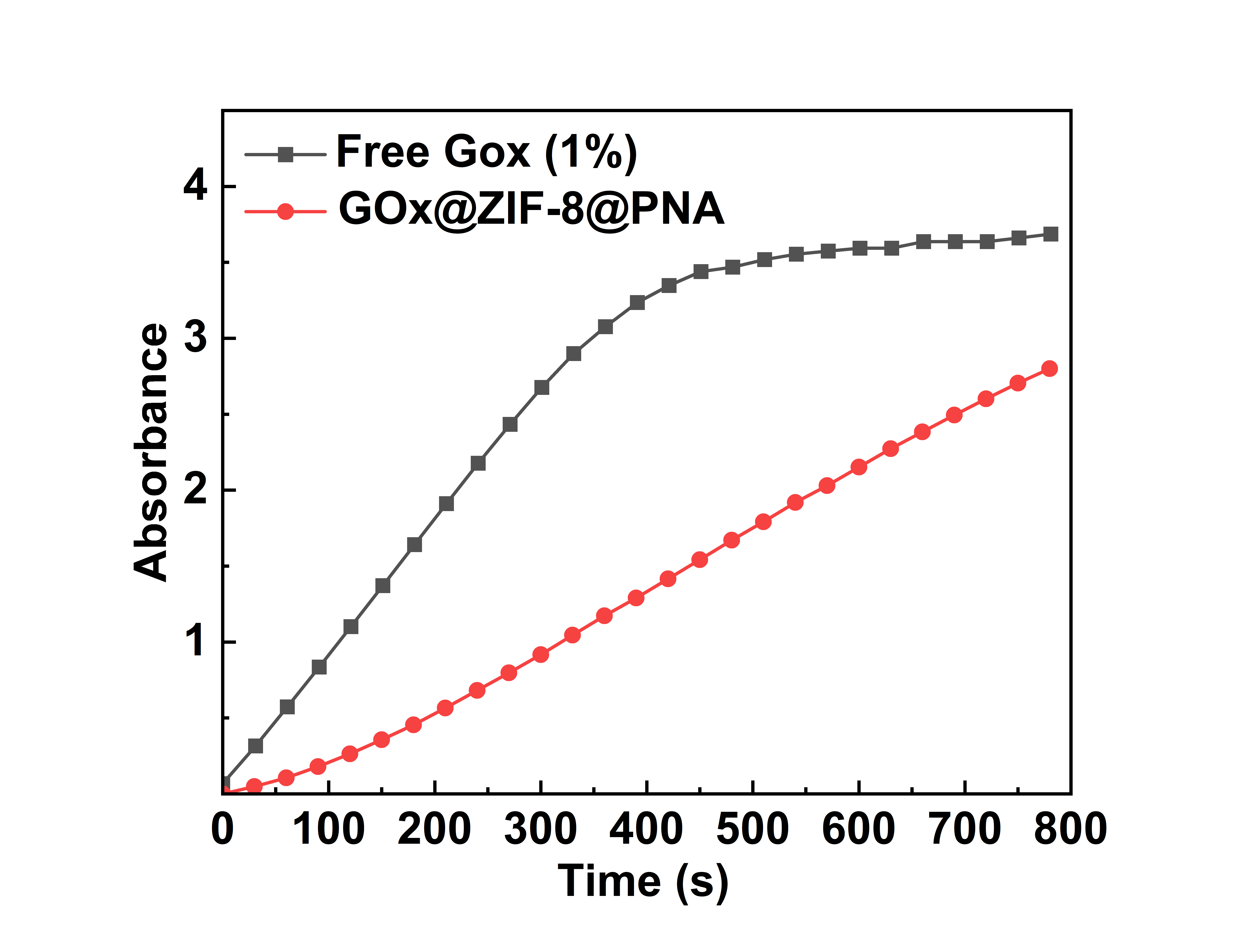


**Figure S18.** The catalytic activity of free GOx and GOx@ZIF-8@PNA, where the concentration of free GOx was 1% of that in the latter.


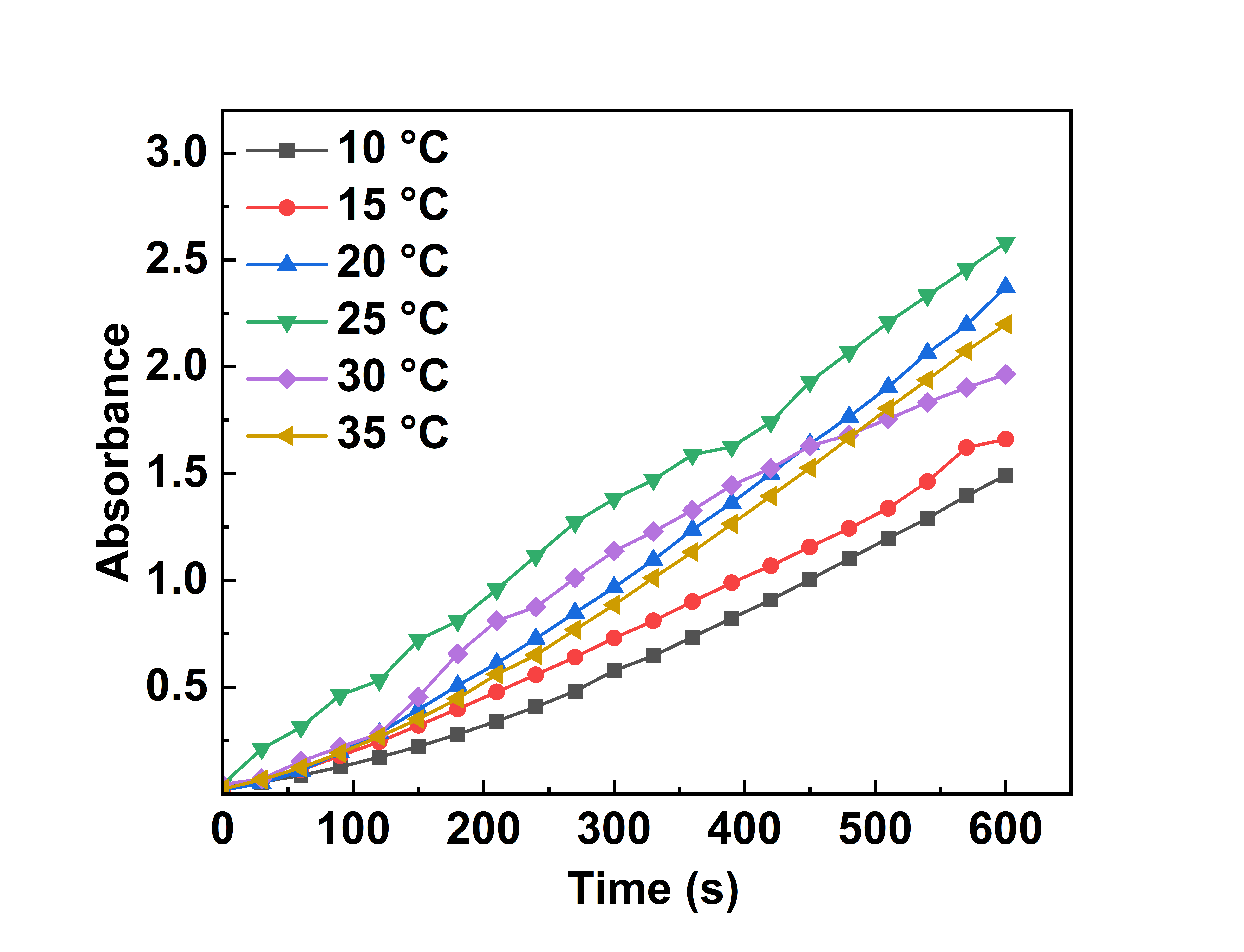


**Figure S19.** Catalytic activity of GOx@ZIF-8@PNA at different temperatures.


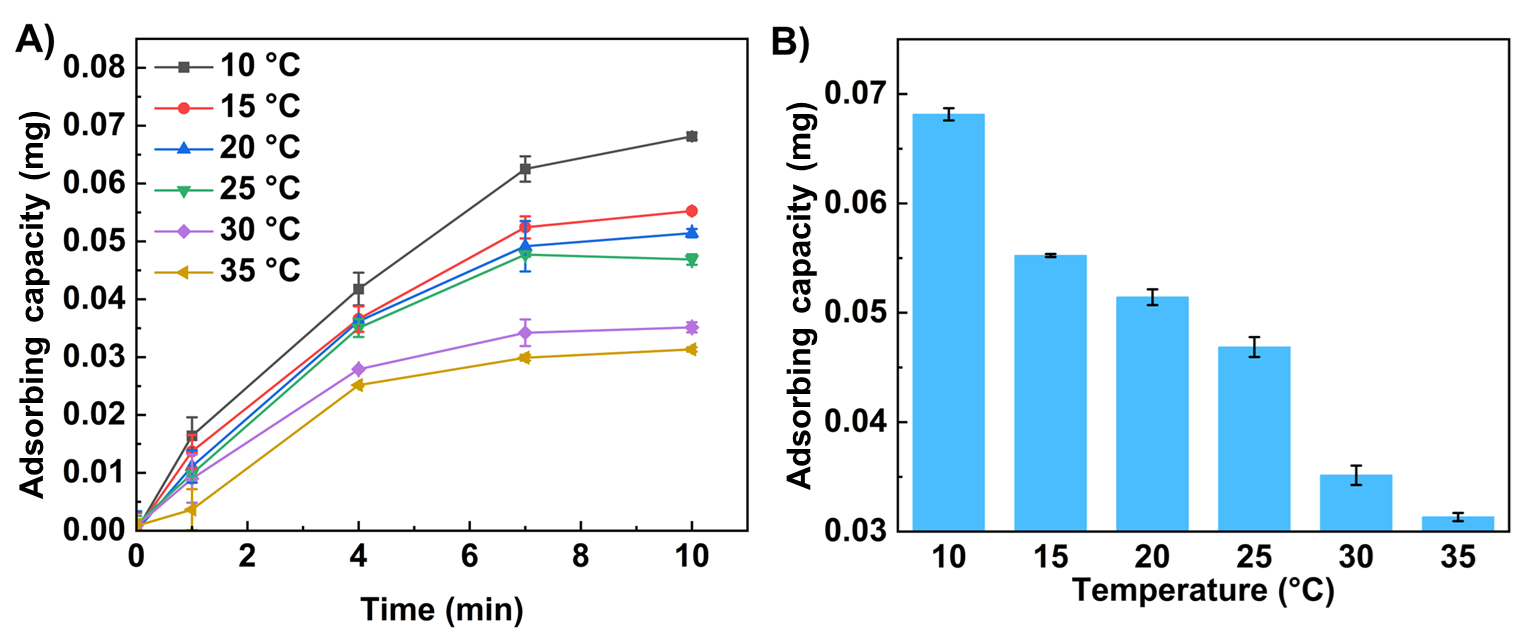


**Figure S20. A)** Adsorption curves of PNA towards MB at different temperatures. **B)** Absorbing capacity of PNA towards MB at different temperatures.


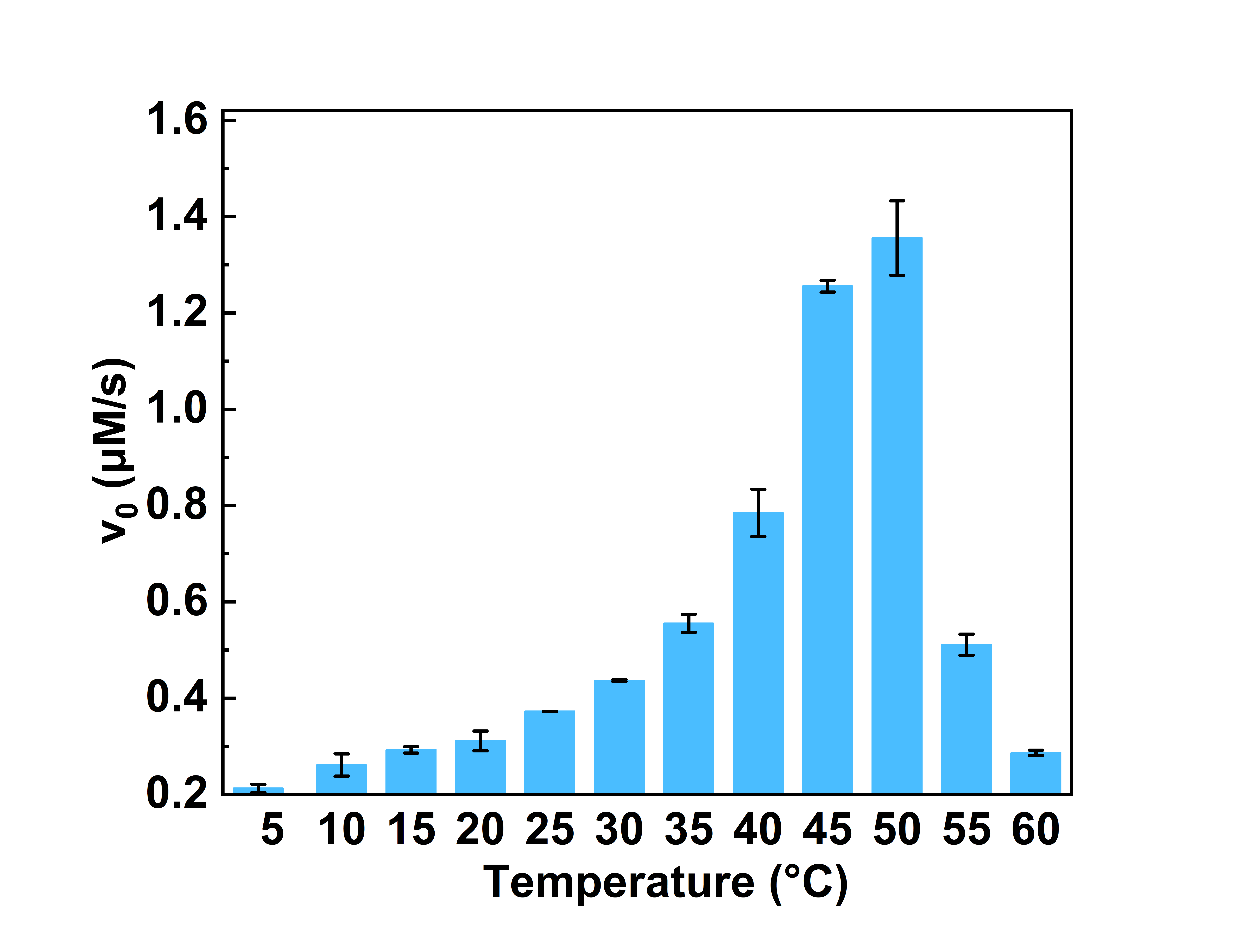


**Figure S21.** The catalytic activity of GOx@ZIF-8 at different temperatures.


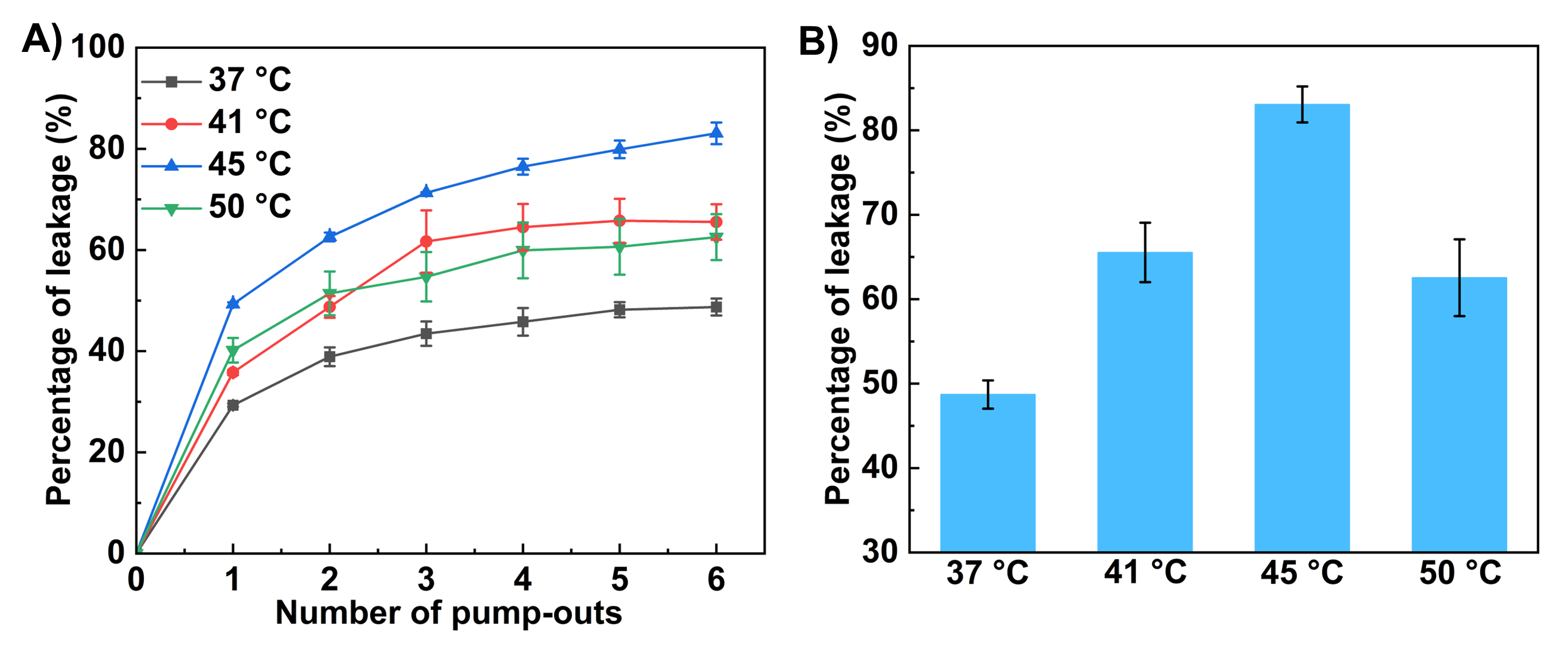


**Figure S22. A)** The release profiles of MB after varying numbers of pump-out cycles at different temperatures. **B)** Percentage release of MB at different temperatures.


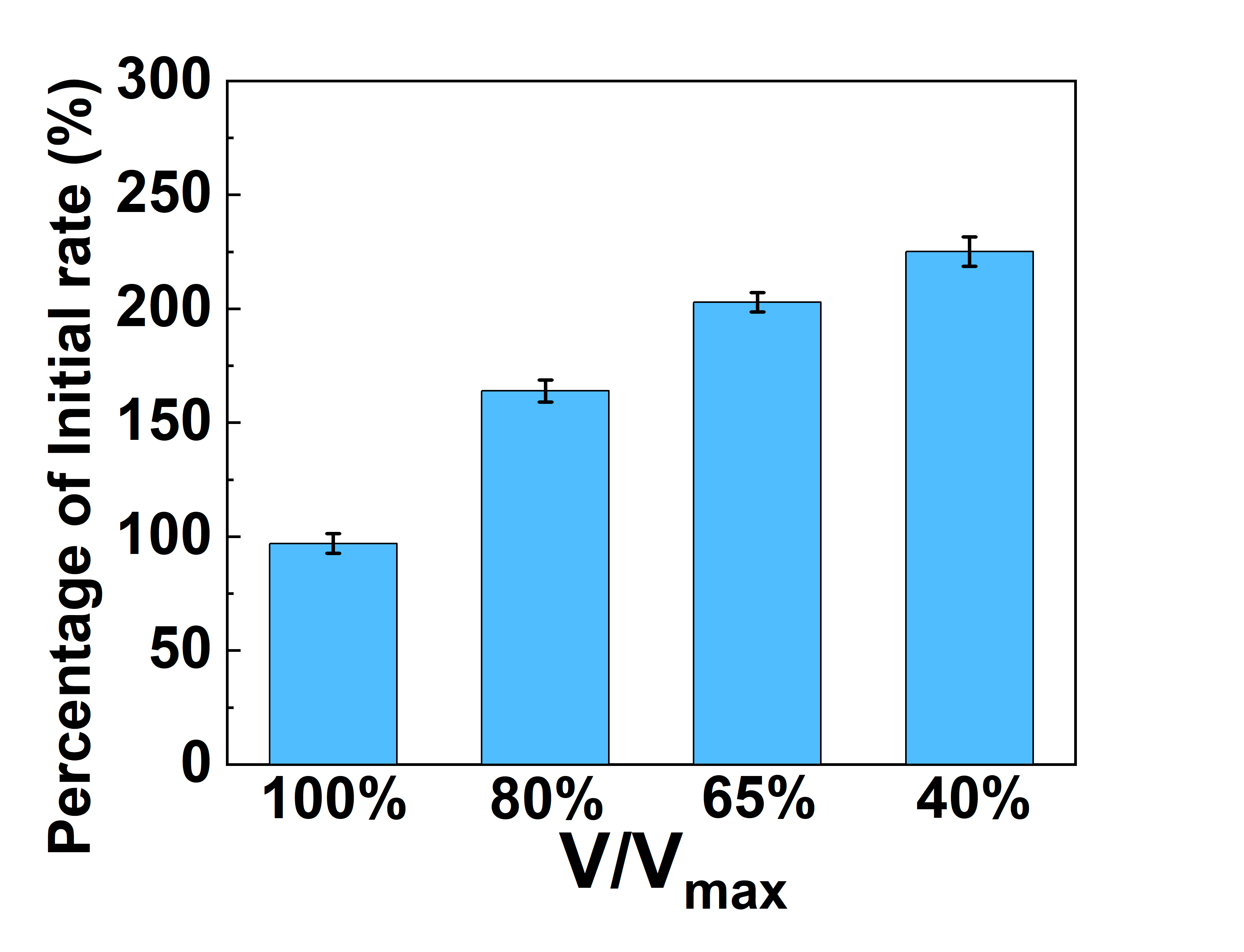


**Figure S23.** The initial catalytic rate of GOx@ZIF-8@PNA versus percentage of gel contraction.


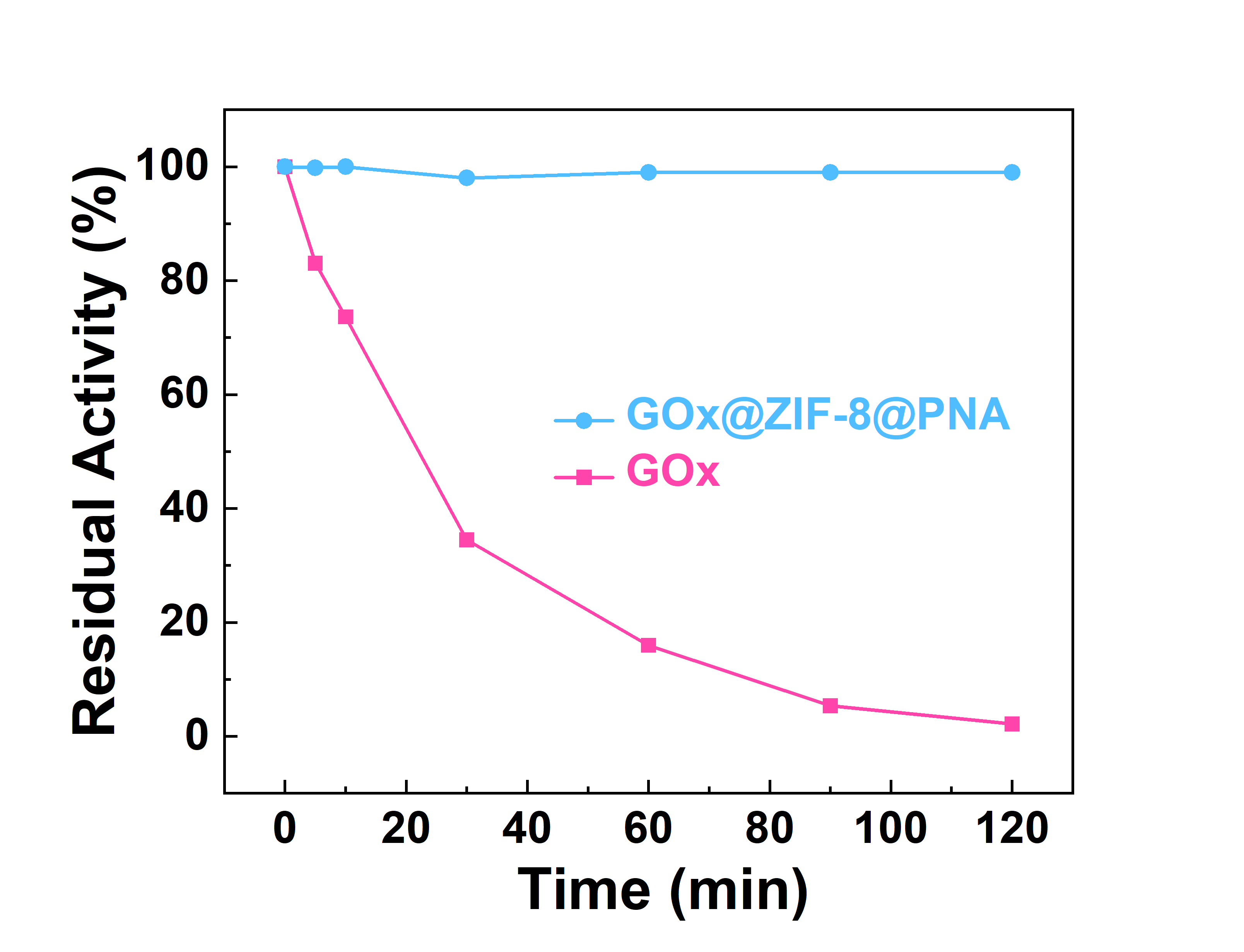


**Figure S24.** Thermostability of GOx and GOx@ZIF-8@PNA over time at 60 ℃.


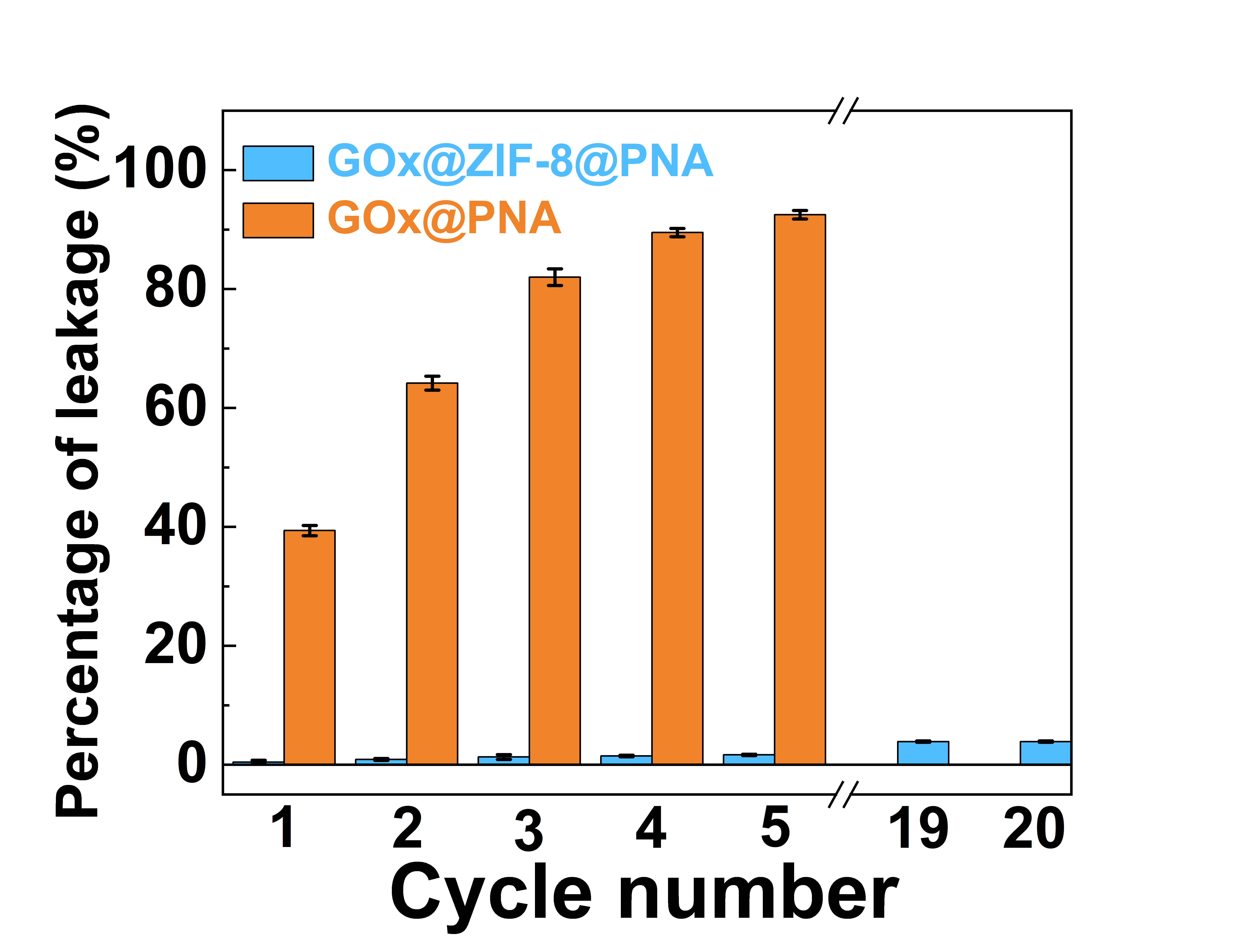


**Figure S25.** The GOx leakage of GOx@ZIF-8@PNA and GOx@PNA during recyclability.


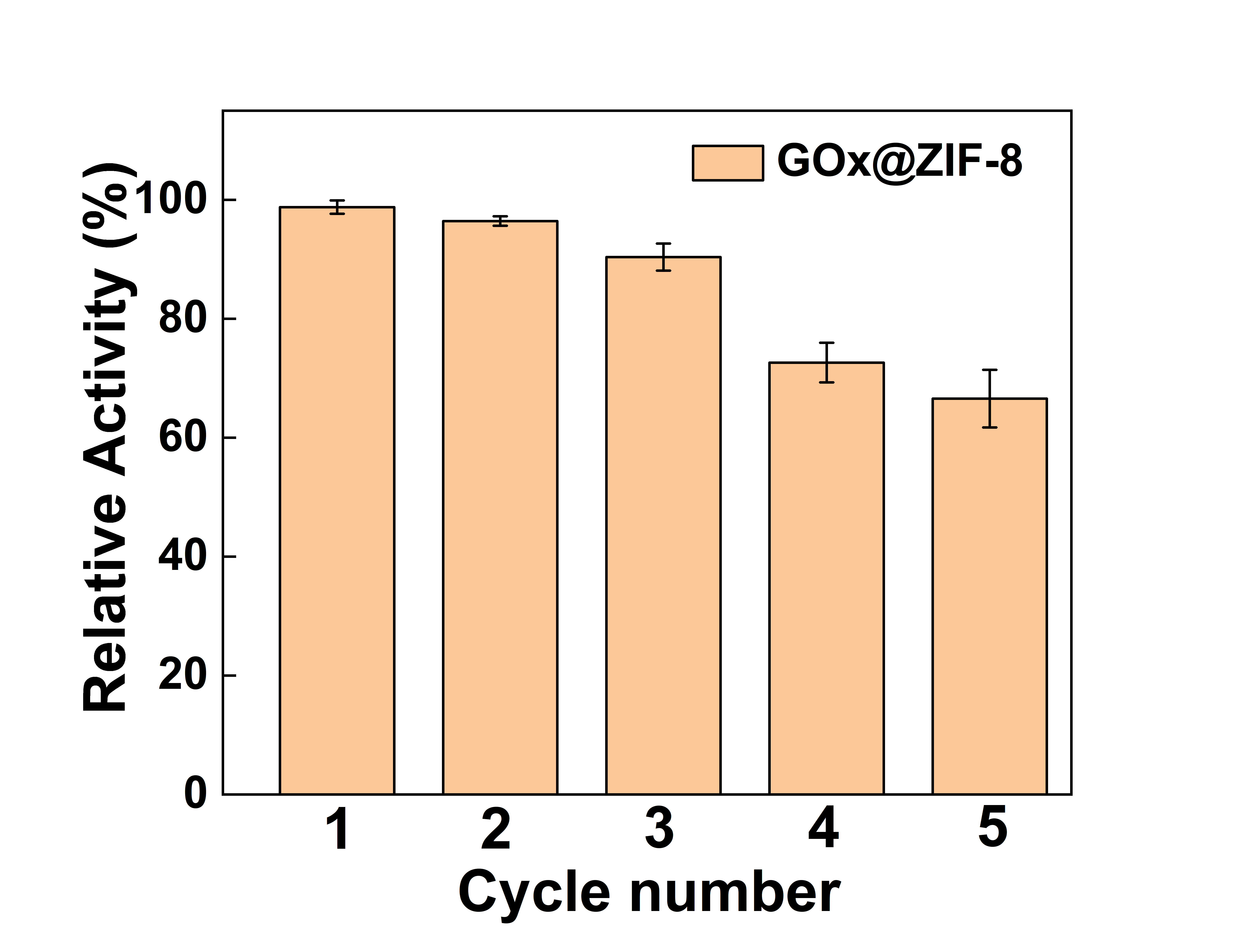


**Figure S26.** Recycling experiments of GOx@ZIF-8.


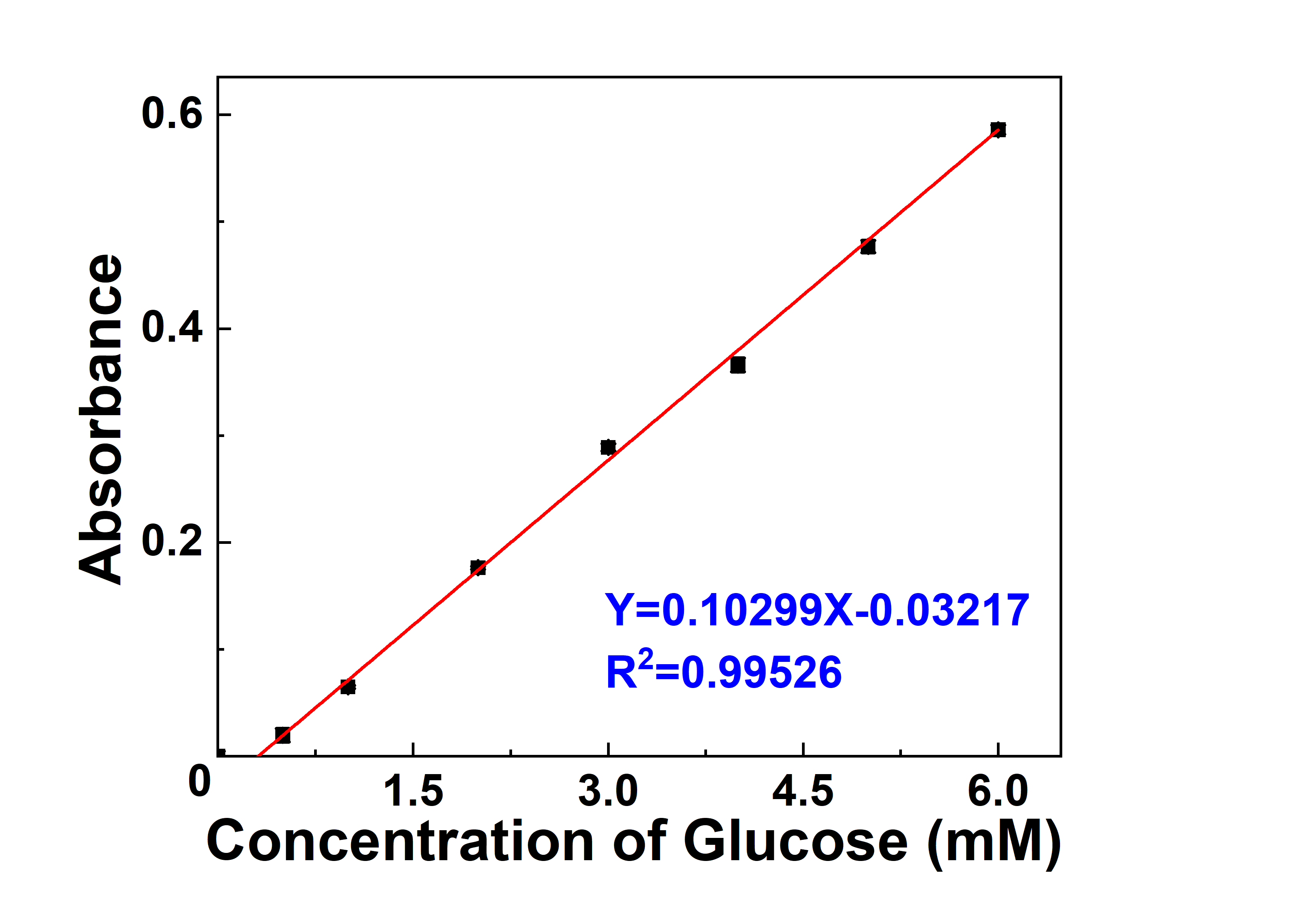


**Figure S27.** Standard curve of glucose aqueous solution based on DNS method.


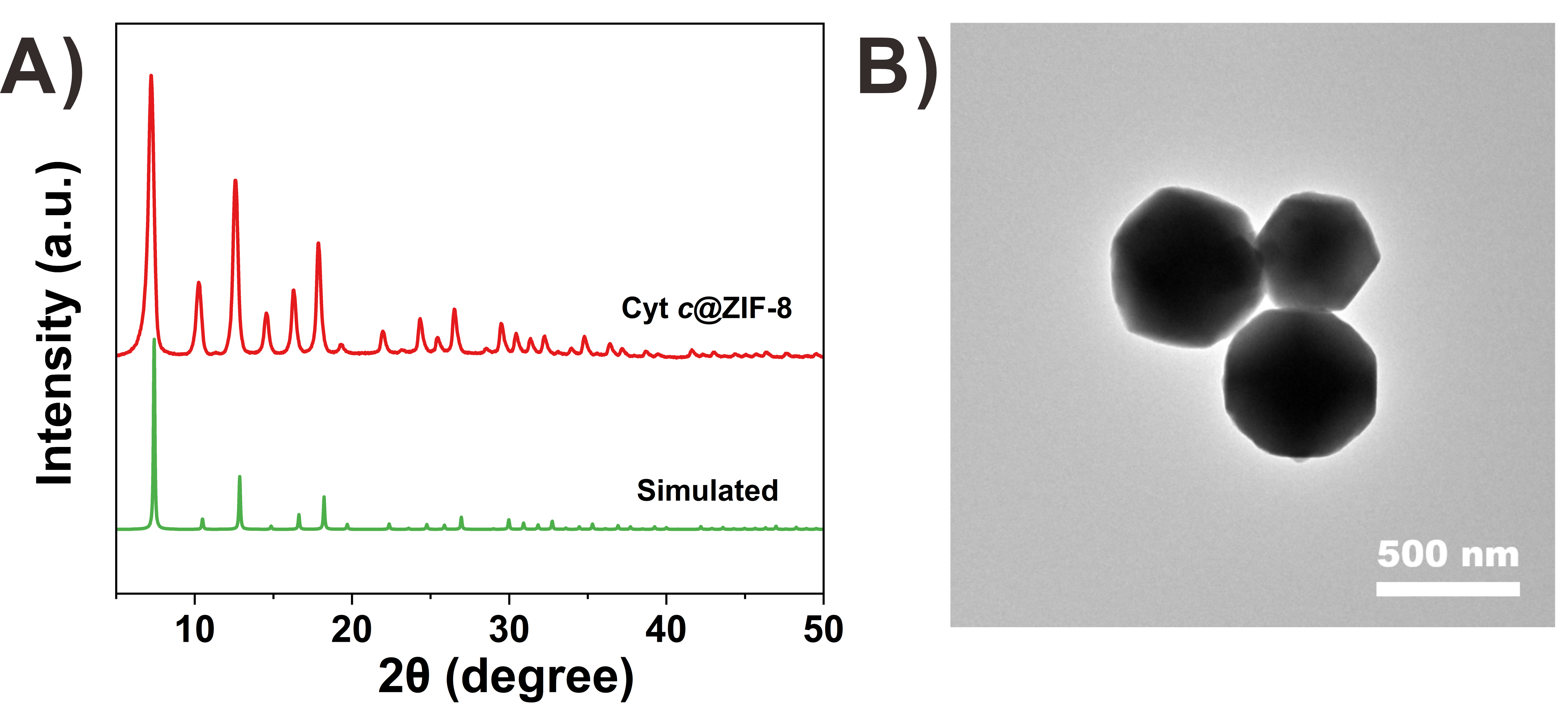


**Figure S28.** **A)** PXRD patterns of Cyt *c*@ZIF-8. **B)** TEM image of Cyt *c*@ZIF-8.


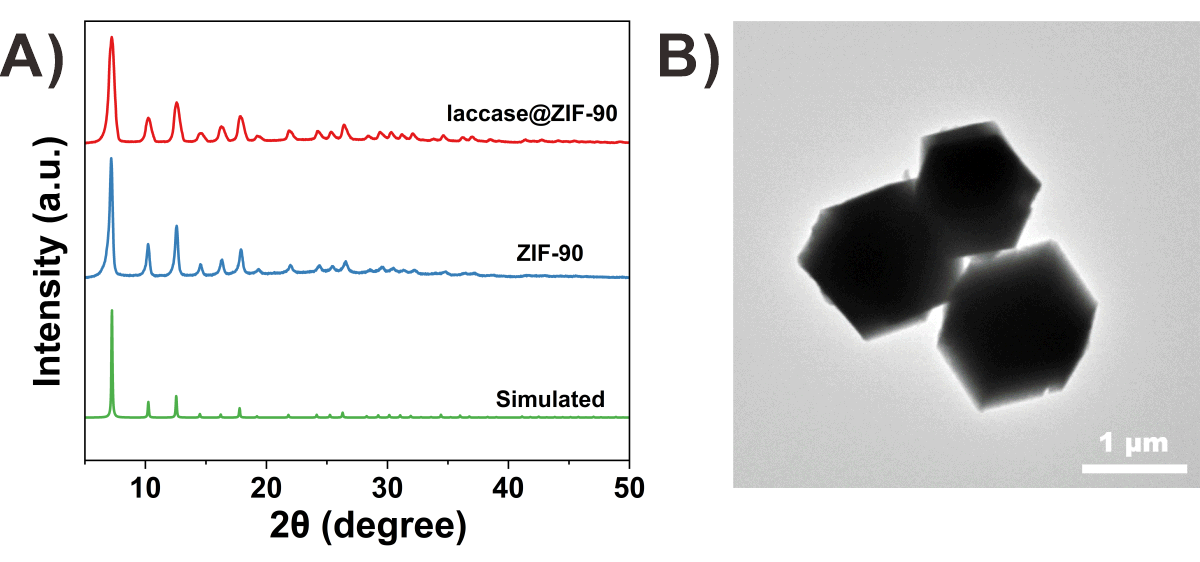


**Figure S29.** **A)** PXRD patterns of ZIF-90 and laccase@ZIF-90. **B)** TEM image of laccase@ZIF-90.


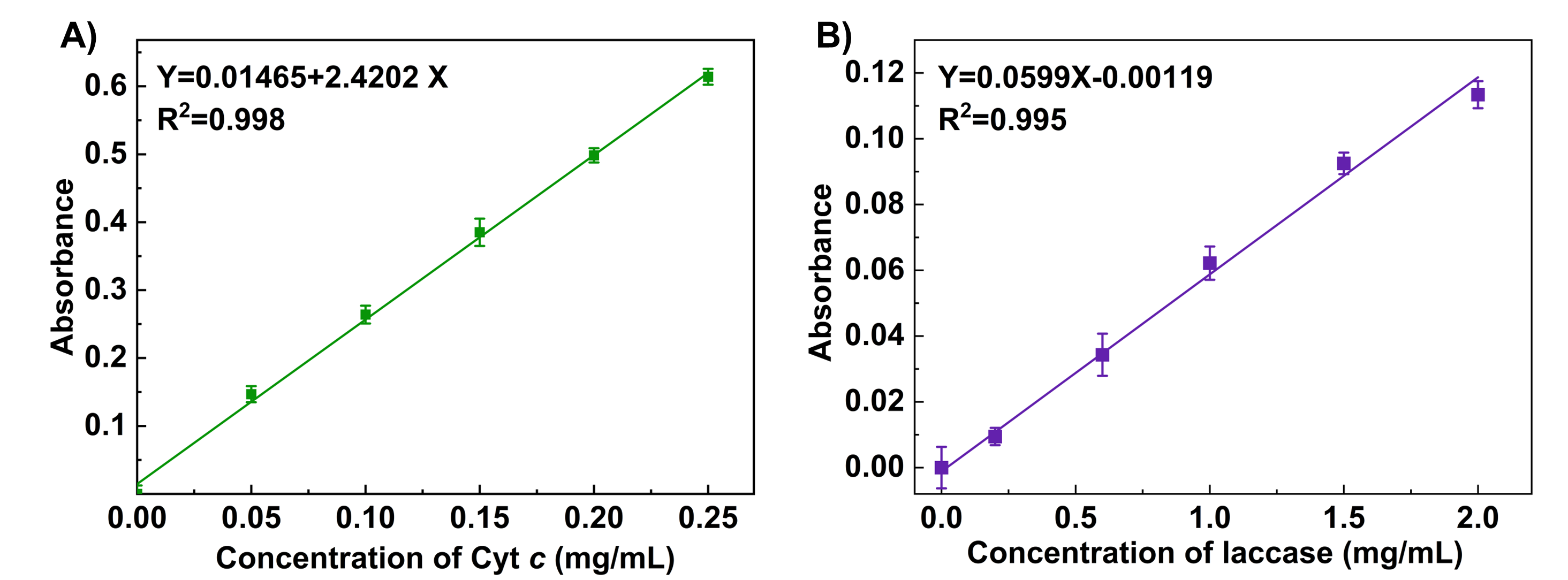


**Figure S30.** Standard curve of **A)** Cyt *c* and **B)** laccase based on Bradford method.


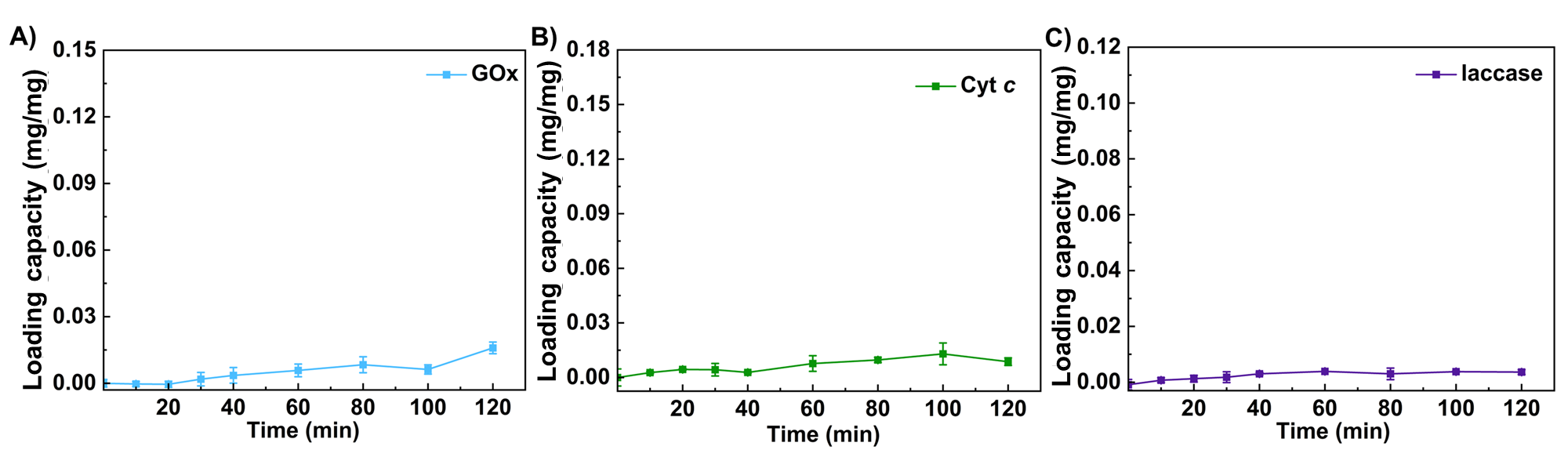


**Figure S31.** Adsorption isotherms of **A)** GOx on ZIF-8, **B)** Cyt *c* on ZIF-8, and **C)** laccase on ZIF-90.


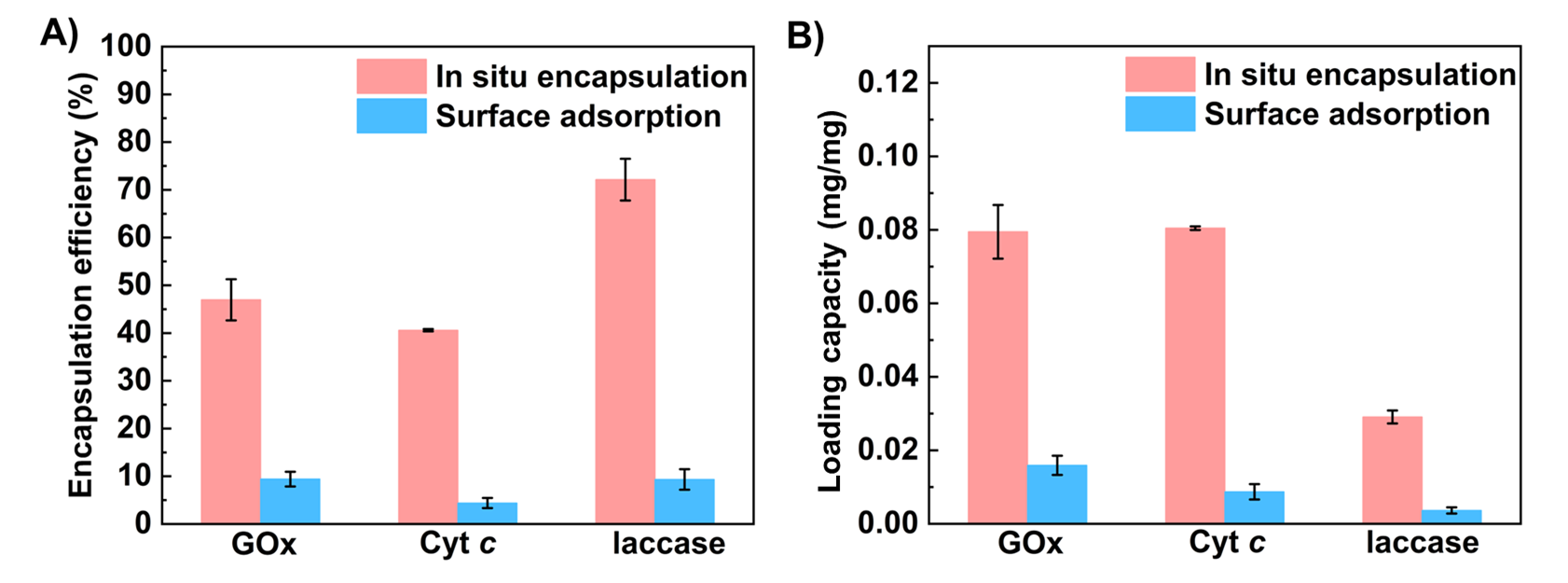

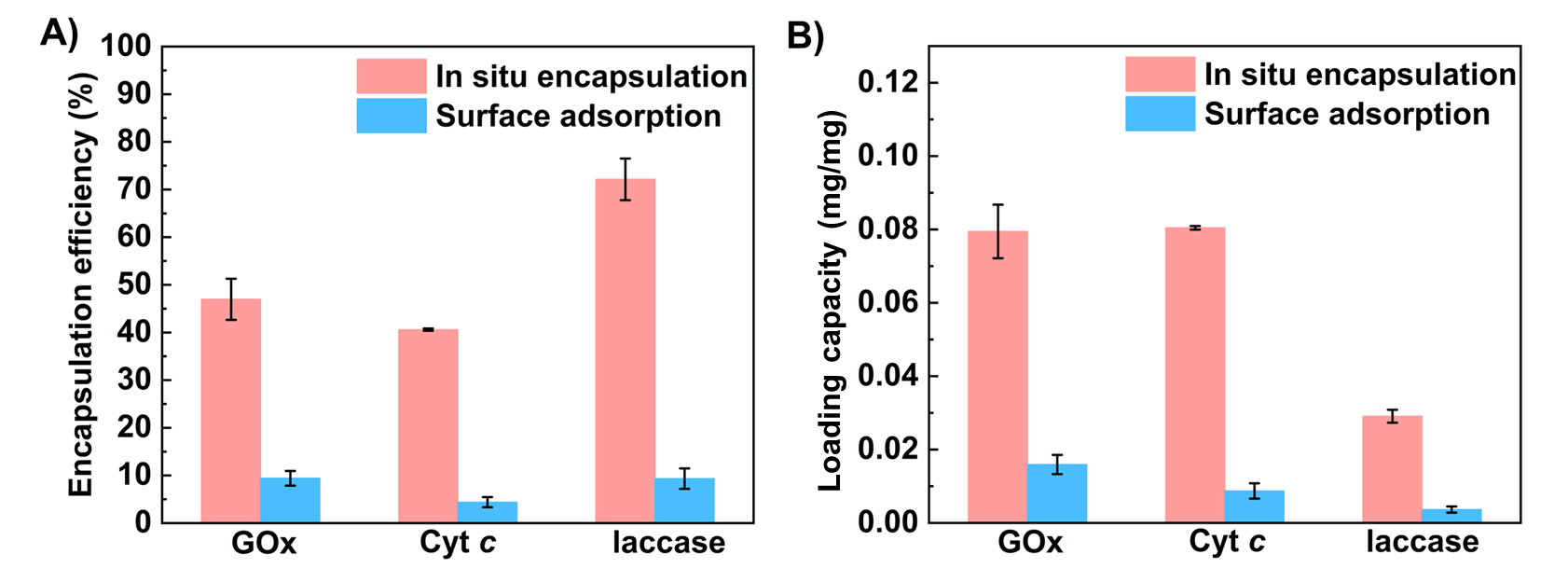


**Figure S32. A)** Encapsulation efficiency and **B)** loading loading capacities of GOx by ZIF-8, Cyt *c* by ZIF-8, and laccase by ZIF-90, as evaluated using both in situ encapsulation and surface adsorption methods.


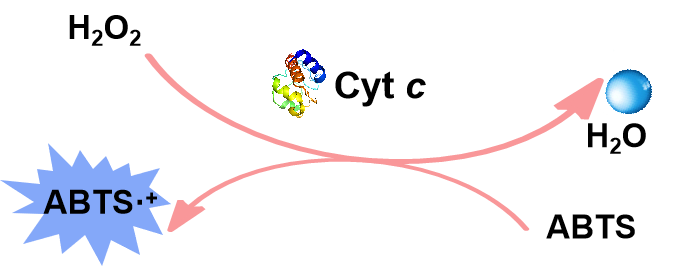


**Figure S33.** Schematic diagram of the principle of Cyt *c*-catalyzed oxidation of ABTS in the presence of H_2_O_2_.


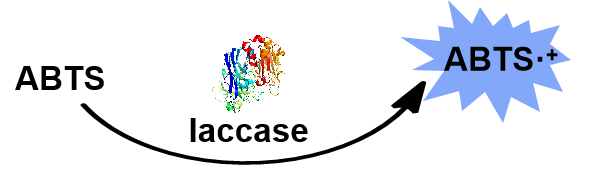


**Figure S34.** Schematic diagram of laccase-catalyzed oxidation of ABTS.


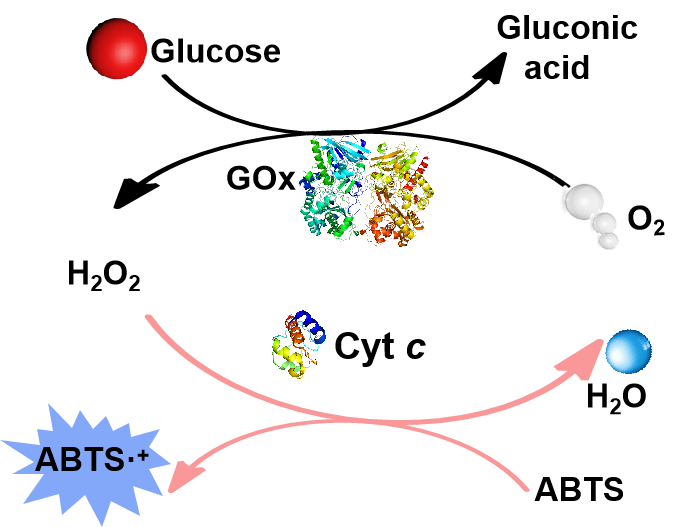


**Figure S35.** Schematic diagram of the mechanism of glucose decomposition and ABTS oxidation catalyzed by GOx/Cyt *c* cascade reaction.


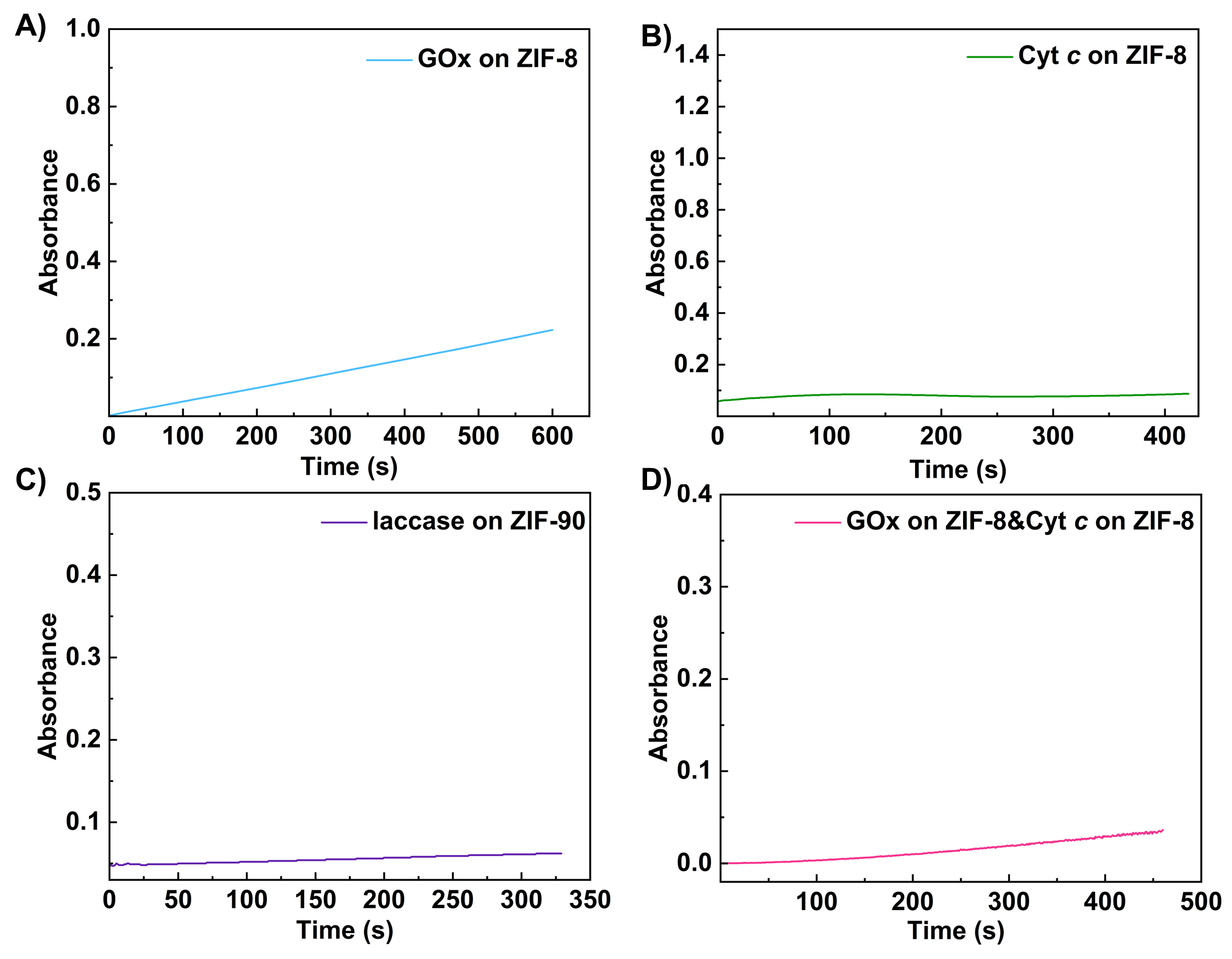


**Figure S36.** Catalytic performance curves of **A)** GOx immobilized on ZIF-8, **B)** Cyt *c* immobilized on ZIF-8, **C)** laccase immobilized on ZIF-90 and **D)** GOx immobilized on ZIF-8&Cyt *c* immobilized on ZIF-8.


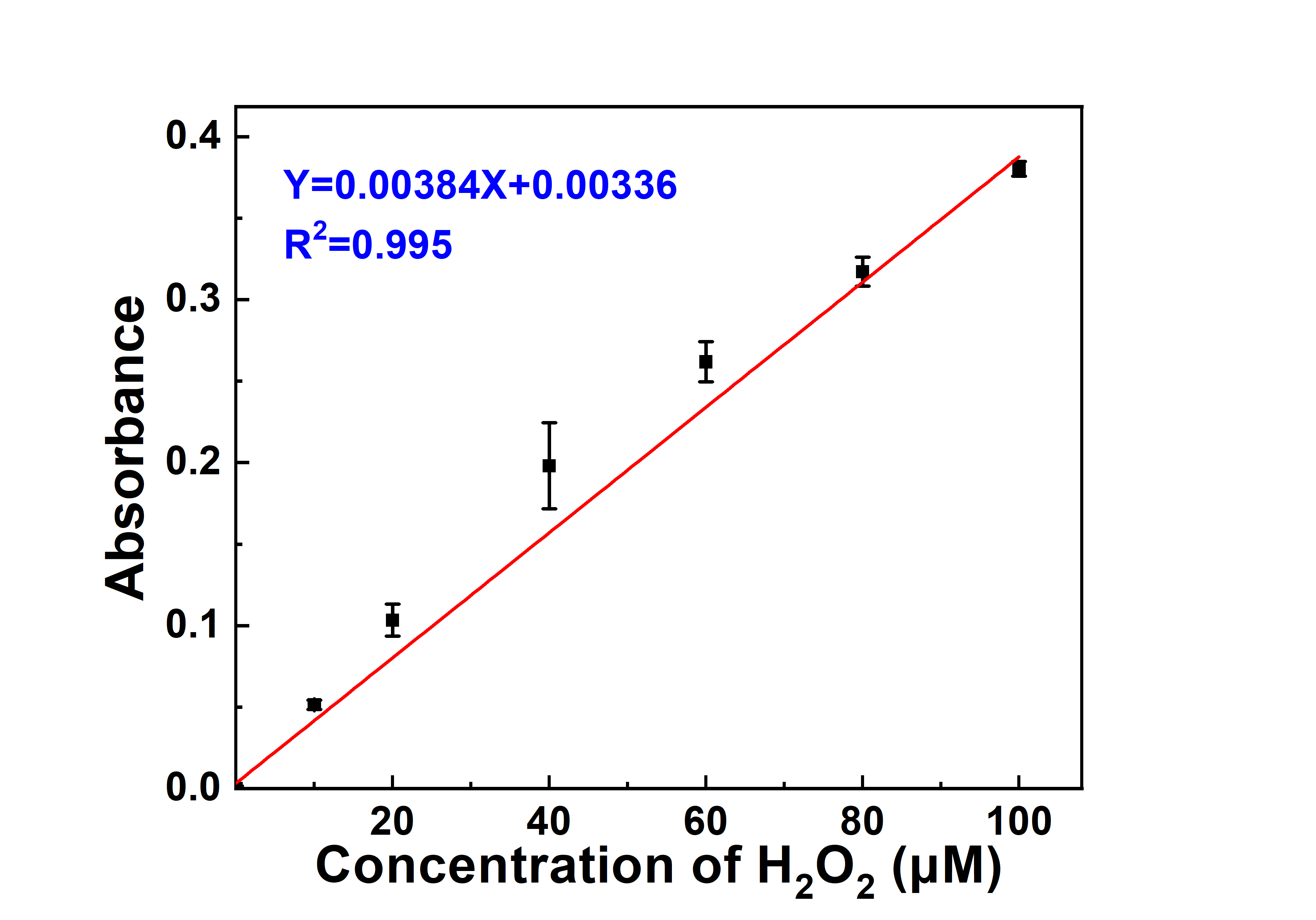


**Figure S37.** Standard curve of H_2_O_2_ based on FOX reagent.


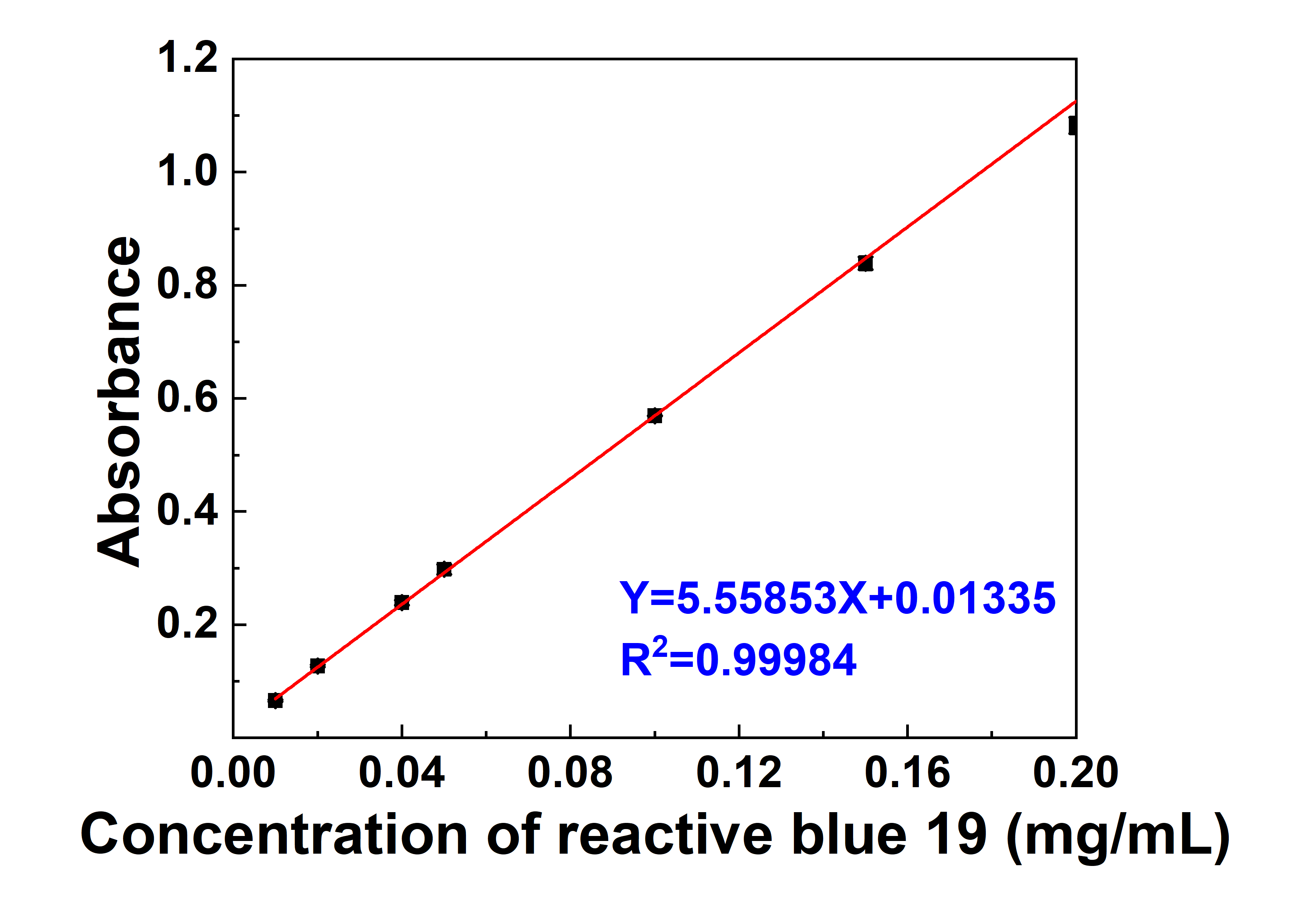


**Figure S38.** Standard curve of reactive blue 19 aqueous solution.


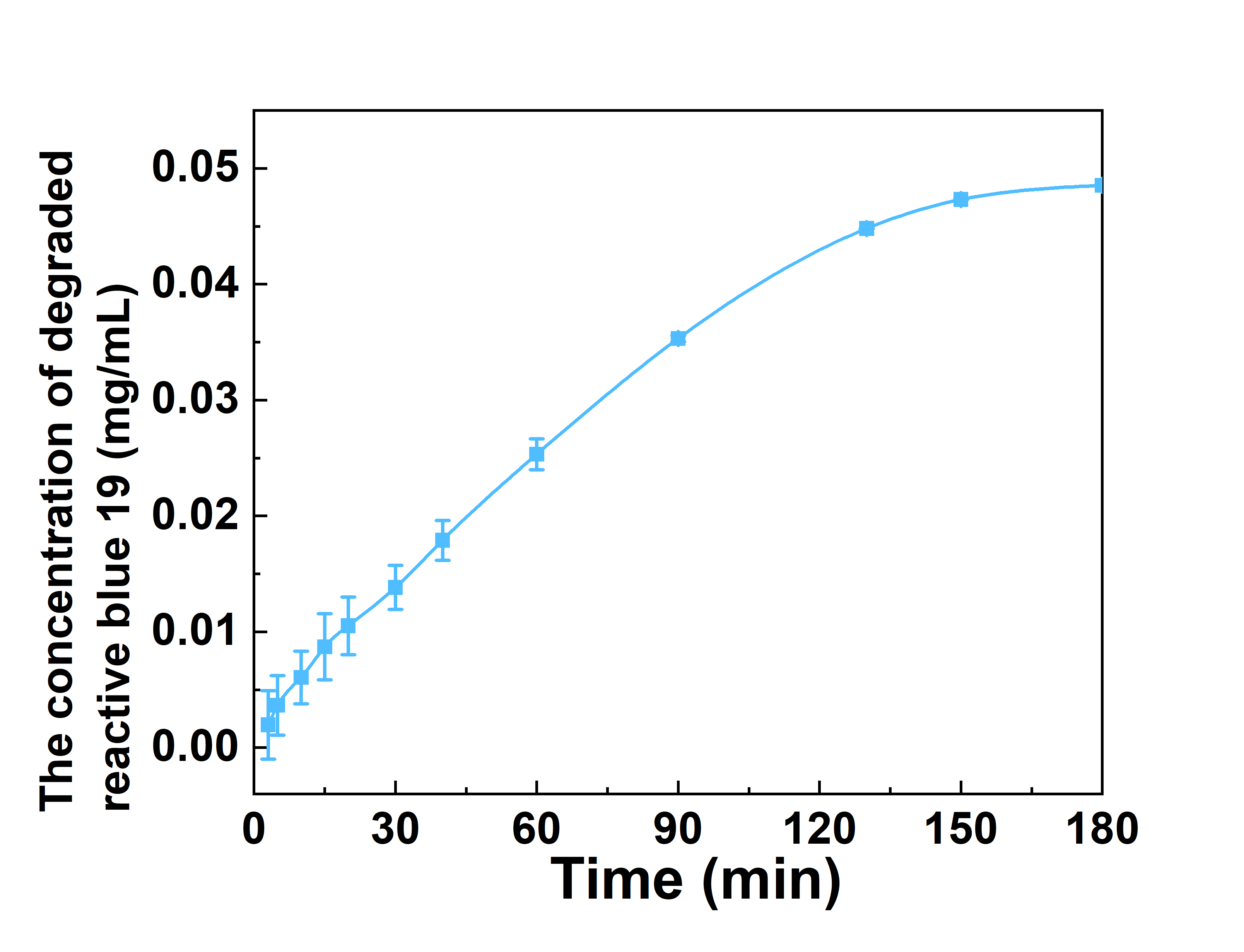


**Figure S39.** Time-dependent degradation of reactive blue 19 by free laccase.

**References**

[1] H. Jung, M. K. Kim, J. Y. Lee, S. W. Choi, J. Kim. Adhesive hydrogel patch with enhanced strength and adhesiveness to skin for transdermal drug delivery. *Adv. Funct. Mater.* **2020**, *30*, 2004407.

[2] M. Li, S. Qiao, Y. Zheng, Y. H. Andaloussi, X. Li, Z. Zhang, A. Li, P. Cheng, S. Ma, Y. Chen. Fabricating covalent organic framework capsules with commodious microenvironment for enzymes. *J. Am. Chem. Soc.* **2020**, *142*, 6675-6681.
